# Unexpectedly High Capacitance of the Metal Nanoparticle/Water Interface: Molecular‐Level Insights into the Electrical Double Layer

**DOI:** 10.1002/anie.202112679

**Published:** 2021-12-17

**Authors:** Mahnaz Azimzadeh Sani, Nicholas G. Pavlopoulos, Simone Pezzotti, Alessandra Serva, Paolo Cignoni, Julia Linnemann, Mathieu Salanne, Marie‐Pierre Gaigeot, Kristina Tschulik

**Affiliations:** ^1^ Analytical Chemistry II Faculty of Chemistry and Biochemistry Ruhr University Bochum 44801 Bochum Germany; ^2^ Schulich Faculty of Chemistry Technion—Israel Institute of Technology 32000 Haifa Israel; ^3^ Physical Chemistry II Faculty of Chemistry and Biochemistry Ruhr University Bochum 44780 Bochum Germany; ^4^ Sorbonne Université CNRS Physico-chimie des Electrolytes et Nanosystèmes Interfaciaux, PHENIX 75005 Paris France; ^5^ Institut Universitaire de France (IUF) 75231 Paris Cedex 05 France; ^6^ Université Paris-Saclay Univ Evry CNRS LAMBE 91025 Evry-Courcouronnes France

**Keywords:** electrical double-layer capacitance, nano-impact electrochemistry, nanoparticles, solid–liquid interface

## Abstract

The electrical double‐layer plays a key role in important interfacial electrochemical processes from catalysis to energy storage and corrosion. Therefore, understanding its structure is crucial for the progress of sustainable technologies. We extract new physico‐chemical information on the capacitance and structure of the electrical double‐layer of platinum and gold nanoparticles at the molecular level, employing single nanoparticle electrochemistry. The charge storage ability of the solid/liquid interface is larger by one order‐of‐magnitude than predicted by the traditional mean‐field models of the double‐layer such as the Gouy–Chapman–Stern model. Performing molecular dynamics simulations, we investigate the possible relationship between the measured high capacitance and adsorption strength of the water adlayer formed at the metal surface. These insights may launch the active tuning of solid–solvent and solvent–solvent interactions as an innovative design strategy to transform energy technologies towards superior performance and sustainability.

## Graphical abstract

Nano‐impact electrochemistry revealed an anomalously high capacitance for Pt nanoparticles and to a lower extent for Au nanoparticles at potentials more negative than their potential of zero charge. MD calculations showed that the strong interaction of metal with water molecules leads to water chemisorption and accumulation of ions in the Helmholtz layer beyond expectation, resulting in a higher interface charge storage ability.

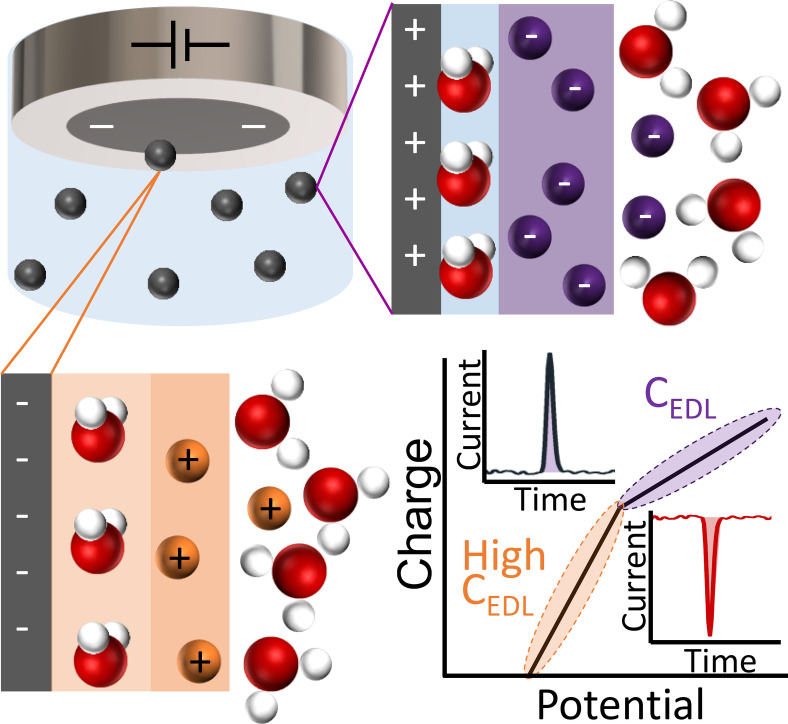

## Introduction

The key to understand and control interfacial electrochemical phenomena such as electrocatalysis and corrosion lies in revealing the molecular structure of solid/liquid interfaces. Many electrochemical processes are governed by the identity, distribution, and interactions of participating species at and near the electrode surface. These species include charged or polarized reactants, intermediates, and products, which are significantly affected by the large electrostatic fields present in the corresponding electrical double‐layer (EDL) at the solid/liquid interface.^[1]^ How reactants, intermediates, and products are surrounded by solvent molecules potentially affects their stabilization, accumulation, orientation, and interaction towards the solid surface. Hence, the dynamic assembly of electrolyte constituents in the solid/liquid interface region directs faradaic as well as non‐faradaic processes. Thus, besides obvious implications for applications based on EDL charging, for example, supercapacitors, EDL characteristics determine vital functions of several technologies. Accordingly, possibilities to utilize insights into double‐layer structures may range from high‐performance fuel cell electrodes to designed formation of the passivating solid‐electrolyte‐interfaces in lithium ion batteries ensuring stable operation.

The first model of the double‐layer structure was developed more than a century ago.^[2]^ Since then, it underwent several modifications to explain experimental results more precisely (a detailed overview is given in the Supporting information).^[3]^ However, no general model exists, yet, that covers all experimental findings. This is due to the fact that the double‐layer structure and its capacitance are controlled by several factors, including electrode material, electrode morphology, the presence of surface adsorbed species, and the interaction and orientation of water molecules at the interface.[^4^, ^5^, ^6^]

To elucidate the effect of these factors, a microscopic understanding of solid/liquid interfaces in the context of experimentally observed electrochemical‐capacitive behavior is required. This particularly concerns nanomaterials since their high surface‐to‐volume ratio is advantageous for many applications. Especially regarding material's design, the intriguing question arises if and how nanoscaling can further affect the intrinsic specific capacitance per area of a material. However, the required explicit characterization of double‐layer capacitance is complicated for nanoparticles: They must be processed to complete electrodes for conventional electrochemical measurements, often including additives and resulting in ensemble effects and uncertainties about the electrochemical active surface area.[^7^, ^8^, ^9^, ^10^, ^11^, ^12^, ^13^] To address this issue, we use single entity electrochemistry as a new and powerful tool to investigate differently shaped platinum (PtNPs) and gold nanoparticles (AuNPs). In the “nano‐impact” method, an enhanced current is measured, which is caused by individual nanoparticles that stochastically collide with an ultramicroelectrode.[^7^, ^8^, ^9^, ^10^, ^11^, ^12^, ^13^] During capacitive nano‐impacts, the EDL at the electrode or at the colliding nanoparticle is perturbed. The resulting charging‐discharging processes give rise to transient current‐time events.[^14^, ^15^, ^16^, ^17^] These signals can be analyzed to gain direct insights into the electronic properties of the impacting particles.[^14^, ^16^]

Herein, we exploit these experiments to extract physico‐chemical information about the EDL structure and capacitance. As the EDL rearrangement is measured while the collision takes place, information on both the colloidal and electrically contacted nanoparticle can be derived. We use non‐spherical, high surface area PtNPs and AuNPs in aqueous solution to demonstrate the versatile applicability of these studies for different nanomaterials in industrially relevant, but still poorly understood contexts. In combination with molecular dynamics (MD) simulations, the gained fundamental insights in EDL formation may open up new strategies to guide the smart and efficient development of new and improved electrodes for future renewable energy technologies. At this, influencing the properties of the solvent adlayer at the solid will be substantially involved, since its interaction with the metal surface and the adjacent solvent layer, and the ions dissolved therein can modulate EDL capacitance.

## Results and Discussion

**Figure 1:**
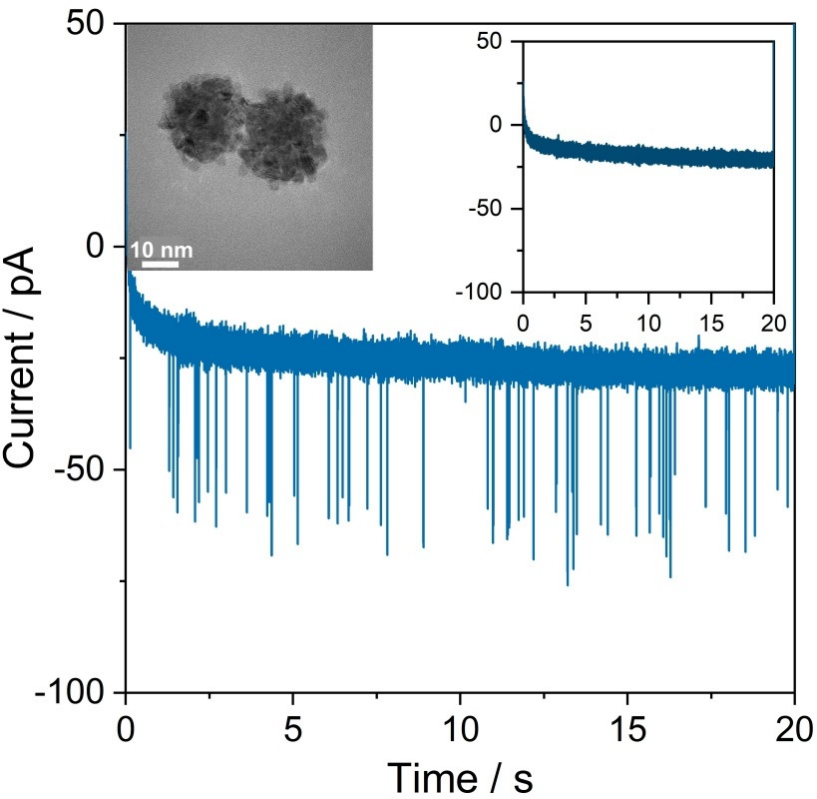
Representative chronoamperogram for nano‐impact measurements recorded at a potential of +0.05 V vs. Ag/AgCl (+0.45 V vs. RHE) in 5.0 mM sodium citrate buffer saturated with Ar in the presence of 30 nm PtNPs. The inset shows the control experiment in the absence of PtNPs.

A carbon fiber ultramicroelectrode was submerged into a 5.0 mM sodium citrate buffer (pH 3.2) saturated with Ar, before 30 nm PtNPs were injected close to the electrode by a micropipette. Immediately after injection, the electrode was potentiostated at +0.05 V vs. Ag/AgCl, 3 M KCl (+0.45 V vs. RHE) for 20 s. Distinct current spikes of about one millisecond duration were observed in the recorded chronoamperogram (Figure 1). Control experiments in the absence of nanoparticles did not contain such features (Figure 1, inlay). This confirms that the spikes are due to charge transfer between a particle and the electrode during the stochastic collision events.

This potential is chosen such that faradaic reactions are avoided (see Figure S3). Hence, the transferred charge reflects the capacitive charging associated with either charging of the impacting nanoparticle or perturbation of the electrode double‐layer by the particle.[^14^, ^17^]

**Figure 2:**
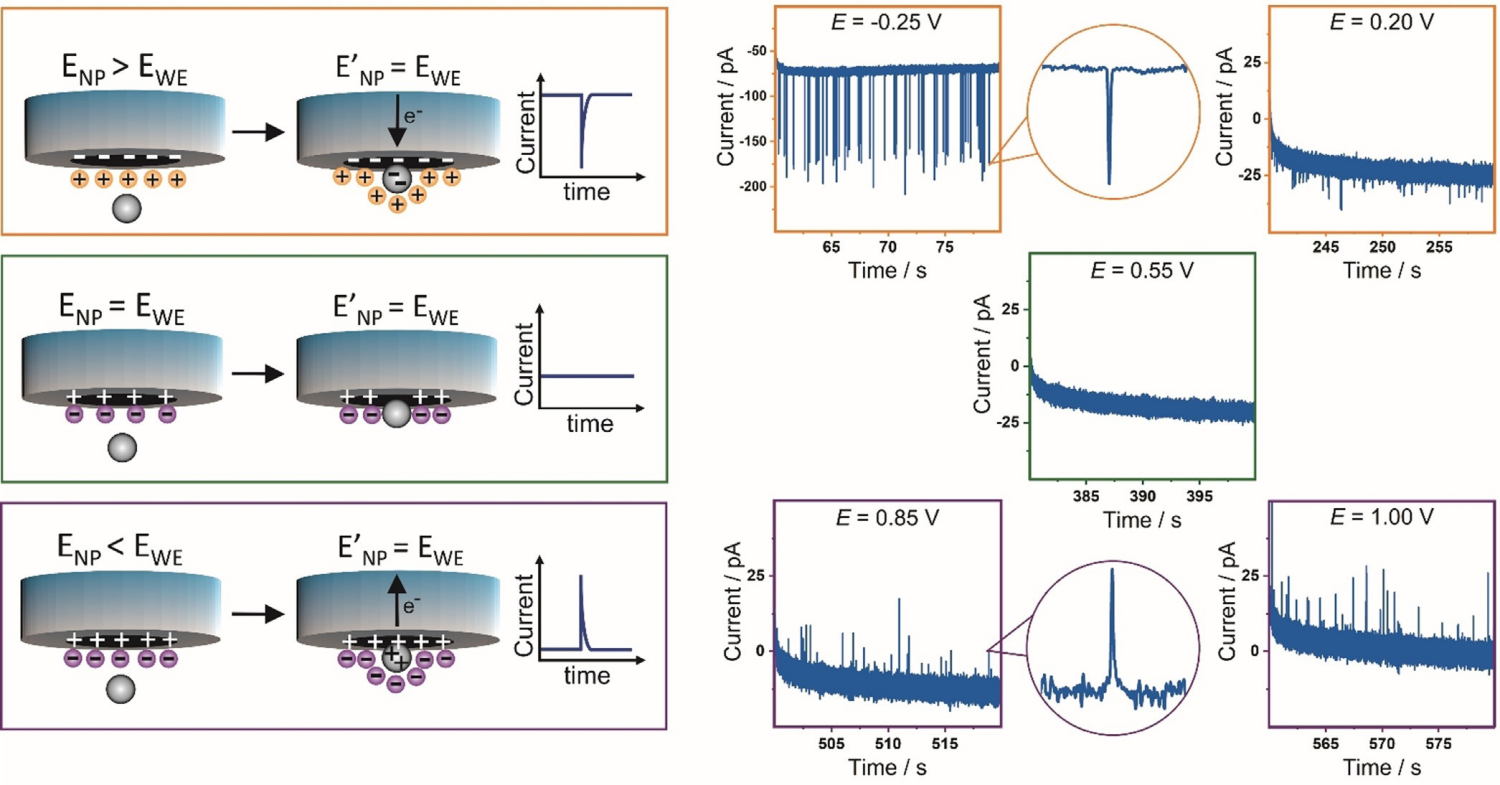
Representation for equilibration of the nanoparticle potential (*E*_NP_) with the working electrode potential (*E*_WE_) upon nanoparticle collision during nano‐impact experiments and representative current–time transients recorded at different applied potentials as mentioned on each graph vs. Ag/AgCl, in 5.0 mM sodium citrate buffer saturated with Ar in the presence of 30 nm PtNPs.

We propose that charge transfer happens because the potential of a nanoparticle in solution is different from that of the electrode. As soon as a nanoparticle establishes electrical contact to the potentiostated electrode, its potential equilibrates to the applied potential (Figure 2).^[18]^ If the potential of the nanoparticle in the solution is more positive than that of the potentiostated electrode, then a flow of electrons from the electrode to the nanoparticle occurs, which results in a negative spike. By applying a more positive potential at the electrode, the impact of the nanoparticle and consequent potential equilibration results in a flow of electrons from the particle to the electrode. In this case, a positive spike is detected. No charge is transferred if the applied potential is equal to the nanoparticle potential before collision that is its intrinsic potential in the solution.

As shown in Figure 2, at low applied potentials, negative spikes are detected, that means, a flow of electrons from the electrode to the NP occurs. Shifting the applied potential to more positive values leads to a steady, nearly linear decrease of the detected negative spike charge, and eventually a change in the sign of the spike.

Furthermore, we ran these experiments using gold and platinum working electrodes to investigate the changes of the transferred charge as a function of the applied potential. If the capacitive spikes are due to perturbation of the electrode EDL, a different behavior should be observed for different electrode materials. Conversely, if the charging of the EDL of the impacting nanoparticles is the origin of observed spikes, identical charge vs. potential behaviors are expected.

**Figure 3:**
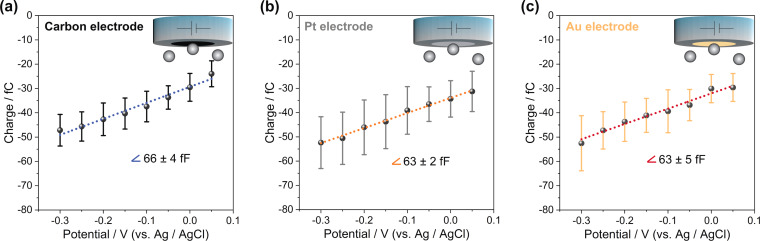
Mean transferred charge vs. applied potential during 30 nm PtNP impacts at a) carbon, b) platinum, and c) gold ultramicroelectodes in 5 mM citrate buffer saturated with Ar; error bars show the standard deviation. (Related chronoamperograms can be found in Figure S4).

To allow a direct comparison, we performed the analysis over a range of potentials, in which the spikes were well‐resolved from the background noise and faradaic reactions were avoided at either of the working electrodes as well as nanoparticles. Hence, we conducted the data analysis over the range of −0.30 V to +0.05 V vs. Ag/AgCl (+0.10 V to +0.45 V vs. RHE). As can be seen in Figure 3, the results for different electrodes: carbon, platinum and gold are nearly identical. Accordingly, we conclude that the observed current spikes originate from (dis)charging the EDL of individual PtNPs while impacting the working electrode and being subjected to the applied potential.

**Figure 4:**
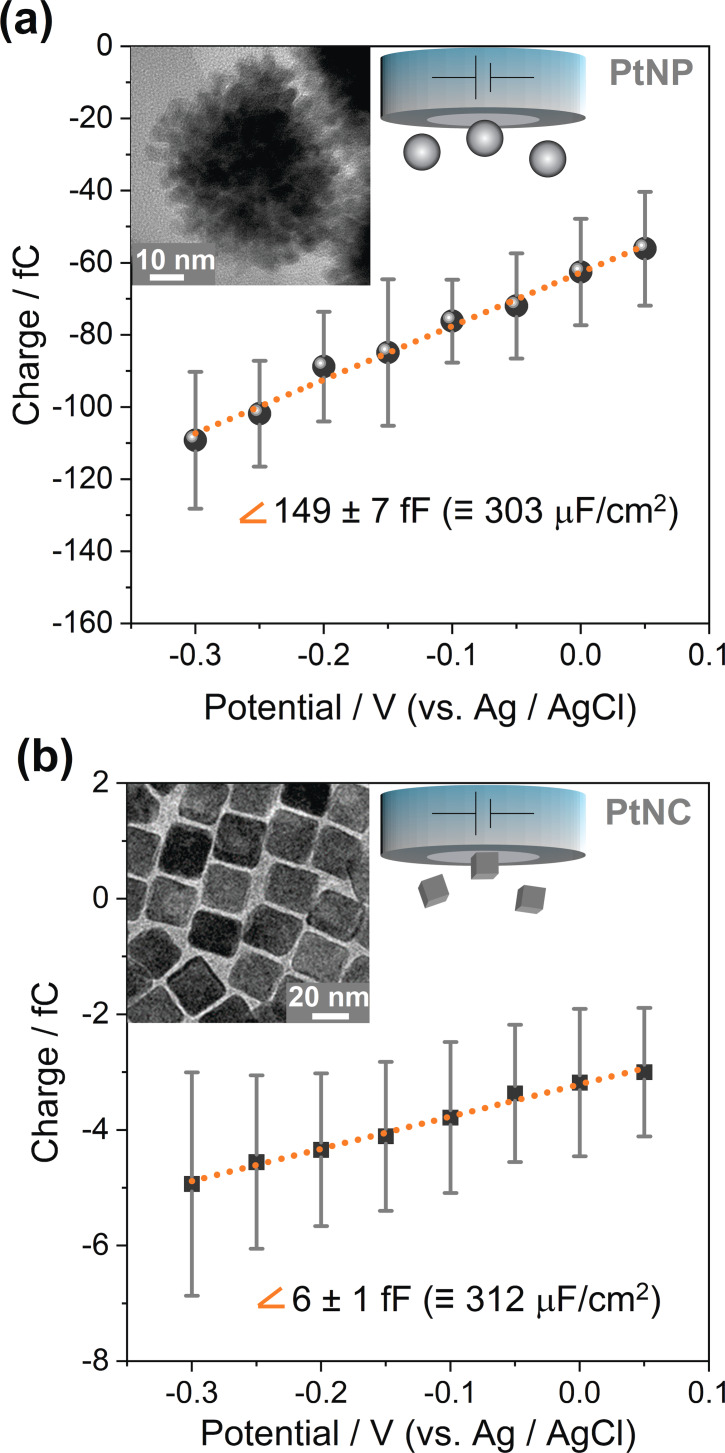
Mean transferred charge vs. applied potential for a) 50 nm raspberry‐like PtNPs and b) 18 nm cubic PtNCs impacting a platinum electrode in 5.0 mM sodium citrate buffer de‐aerated with Ar; error bars represent the standard deviation. Insets show TEM images of the respective nanoparticles (Related chronoamperograms can be found in Figure S8).

As derived in the Supporting Information, the slope of the charge vs. potential plot represents the differential capacitance of the PtNPs’ EDL, which is equal to a specific capacitance of about 300 μF cm^−2^. This is about 15 times higher than the values usually reported for single crystal platinum (20 μF cm^−2^).^[19]^ Such a high specific capacitance for metal‐water interfaces demonstrates a large divergence from the classical models for the EDL (discussed in the Supporting Information) as well as from typical results of classical molecular dynamics.[^5^, ^20^] This may originate from porosity or curvature effects^[21]^ (considering the mesoporous structure of the used raspberry‐like nanoparticles evidenced by TEM, Figure S5), or from specific adsorption effects at the surface of the nanoparticles. To elucidate this issue, we next studied particles of different sizes and morphologies. This was done for nominally 50 nm raspberry‐like PtNPs and 18 nm (edge length) cubic nanoparticles (PtNCs) using a platinum working electrode (Figure 4). As it is summarized in Table 1, an almost identical capacitance value is obtained for the different nanoparticles. This highlights that the observed large value of the EDL capacitance is neither caused by the morphology nor porosity of the nanoparticles.

**Table 1:** Summary of size, measured charge vs. potential slope and capacitance of the used nanoparticles (mean±standard deviation).

Nanoparticle	Surface area [cm^2^]^[a]^	Slope [fF]	Capacitance [μF cm^−2^]
PtNP 30 nm	2.1(±0.4)×10^−10^	63±2	301±61
PtNP 50 nm	4.9(±1.1)×10^−10^	149±7	303±67
PtNC 18 nm	1.9(±0.2)×10^−11^	6±1	312±66

[a] Details regarding the calculations of the nanoparticles’ surface area can be found in the Supporting Information.

Capping agents, which show strong specific adsorption to nanoparticles,^[22]^ might also contribute to the measured high capacitance. However, the very similar capacitances measured for citrate‐capped PtNPs and 3‐mercaptopropionic acid‐capped PtNCs indicate that the nature of these capping agents has no major influence on the capacitance (structure formulae of both capping agents are shown in the Supporting Information).

It should be also noted that, although reduction of native surface oxides may contribute to the absolute measured charge, an interference with the determined capacitances is not expected. This is an electrochemical process rapidly completed when applying sufficient potentials and, hence, its contribution would be constant in the whole investigated potential range, not affecting the measured charge differences upon varied potential steps. Moreover, as it has been shown in Figure S3, hydrogen under potential deposition (H‐UPD) could not be detected on the PtNPs in the citrate buffer, possibly due to the strong specific adsorption of citrate anions on Pt.^[22]^ Therefore, H‐UPD also cannot interfere with the determined capacitances.

**Figure 5:**
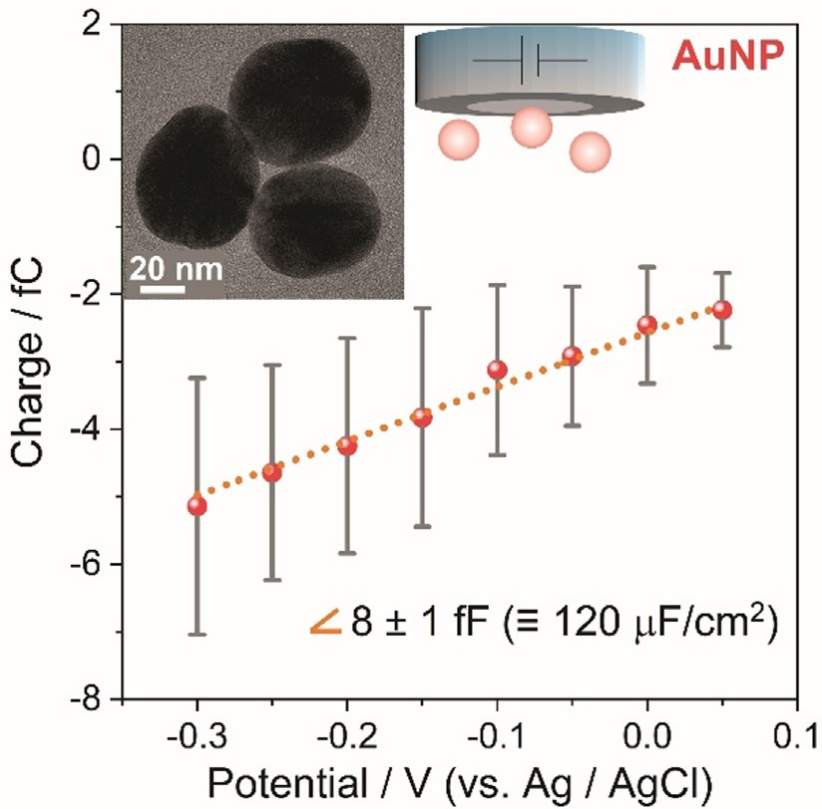
Mean transferred charge vs. applied potential for 46 nm spherical AuNPs impacting a platinum electrode in 5.0 mM citrate buffer de‐aerated with Ar; error bars represent the standard deviation (Related chronoamperograms can be found in Figure S8).

Having ruled out other alternatives, the one order‐of‐magnitude deviation of the EDL capacitance from the values predicted by classical Helmholtz or GCS models is likely attributable to specific interactions of the solvent with the platinum surface. As can be seen in Figure 5, utilizing AuNPs instead of PtNPs under otherwise identical experimental conditions, we measured a capacitance of 120±30 μF cm^−2^. This demonstrates capacitances significantly exceeding classical assumptions also for gold in aqueous electrolytes, but roughly half of the value measured for platinum. A similar conclusion has been reached from recent cyclic voltammetry experiments performed by Koper et al.^[6]^ at macroscopic single crystal electrodes. They reported anomalously high capacitance values for platinum, and to a lower extent for gold single crystals. The authors proposed that these capacitance enhancements resulted from increased ion accumulation in the Helmholtz layer beyond purely electrostatic considerations, which leads to an appreciably more efficient electrostatic screening of the surface charge than predicted by mean‐field theories. Merging these previous results with our experimental findings, thus, suggests that high capacitances are not specific to NPs.

**Figure 6:**
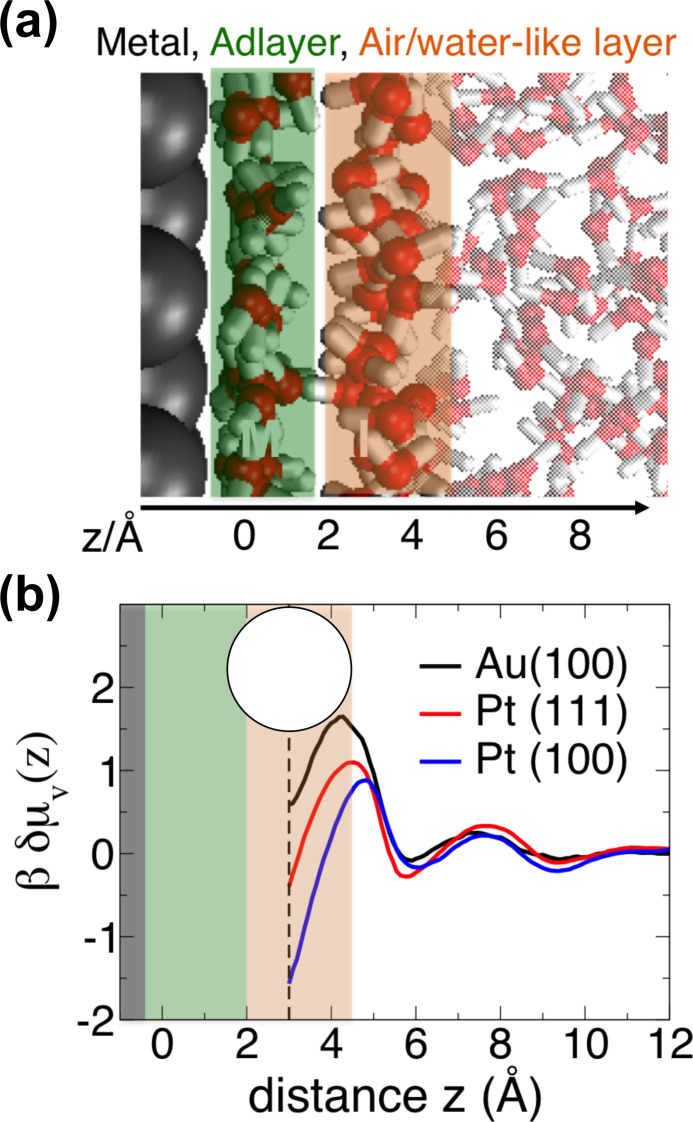
a) MD snapshot illustrating the characteristic water configuration above Au(100) surface. The adlayer (adsorbed on top of the gold surface) and the adjacent air/water‐like layer are highlighted in green and orange, respectively. b) The excess free energy to form a cavity of 3 Å radius as a function of distance away from the Pt(111), Pt(100) and Au(100) surfaces. The dashed line defines the distance of closest approach of the 3 Å cavity to the adlayer and the circle illustrates the cavity's shape (not of real dimension). Platinum profiles are reproduced from Ref. [5].

In the following, we examine the role of the water networks formed in contact with Pt and Au for possibly promoting increased ion concentrations in the Helmholtz layer by means of classical MD simulations (computational details can be found in Supporting Information and Figure S10). The properties of the water adlayer on a metal surface result from a complex balance between metal‐water and water‐water interactions. As proposed by Limmer et al.^[5]^ for platinum/water interfaces and shown here for a gold/water interface (see Figure 6 (a) for an illustration), strong binding of water molecules to the metal surface leads to an ordered water adlayer (highlighted in green in the Figure), which has unfavorable interactions with the adjacent water layer (denoted air/water‐like layer in the Figure, see also Ref. [23]). Adlayer water molecules preferentially orient with their oxygen atoms pointing towards the metal surface and the hydrogen atoms being parallel to it, resulting in a lack of donor sites for hydrogen bonding with the adjacent water layer. This induces local hydrophobicity at the water‐water interface between adlayer and air/water‐like layer, exhibiting enhanced water density fluctuations.[^5^, ^24^, ^25^] The magnitude of water density fluctuations depends on the strength of the water adlayer binding to the metal surface regulated by the commensuration between the water network and the distance and size of atoms forming the metal surface. It can be evaluated from the simulations by monitoring the number of water oxygen centers within an observation volume, here a 3 Å radius sphere (see Methods section). The probability to find zero oxygen centers within the sphere defines the free energy cost to form a cavity in the liquid (*δμ_v_* (*z*)). Figure 6 (b) compares the *δμ_v_* (*z*) profiles as a function of the distance (*z*) between the cavity center and the adlayer in contact with Pt(100), Pt(111) and Au(100) surfaces. The minimum value for the free energy cost to form a cavity at *z=*3 Å, that is, right after the adlayer, indicates that the adlayer is almost disconnected from the adjacent air/water‐like layer, while being strongly bound to the metal. The lower *δμ_v_* (*z*) value in the minimum found for platinum with respect to gold indicates that this effect is amplified at platinum/water interfaces. It demonstrates that the binding strength of the water adlayer to the surface and hence its hydrophobicity towards the adjacent water layer are more pronounced on platinum than on gold. This conclusion is in line with the binding energies of a single water molecule on these metals as estimated by Michaelides et al.^[26]^

The cavities formed in the liquid can accommodate small solutes and ions. In case of ions (or other hydrophilic solutes), the total solvation free energy may be decomposed into two steps:^[27]^ (i) creating a cavity in the liquid, with an associated free energy cost to distort the water hydrogen‐bond network and (ii) filling the cavity with the ion, with an associated free energy gain due to favorable ion‐water interactions. Solvation free energy is then obtained as the sum of these two free energy terms. Larger water density fluctuations can lower the free energy cost of step (i) by increasing the probability of cavity formation in the liquid, thus favoring ion adsorption.[^5^, ^24^, ^28^] Accordingly, the stronger binding of the water adlayer at platinum vs. gold can lead to increased ion accumulation in the Helmholtz layer region and thus, the larger capacitances measured for platinum.

So far, classical MD simulations only reported capacitances in the range of 5–10 μF cm^−2^ for aqueous electrolytes.^[5]^ However, a very recent study on gold‐aqueous sodium chloride interfaces demonstrates the influence of the parameterization of the electrostatic parameters on the electrode/electrolyte properties and shows that larger differential capacitances up to about 125 μF cm^−2^ can be obtained for strongly metallic electrodes by changing the width of the Gaussian charge distribution used to represent the atomic charges of the gold surface.^[29]^ The high capacitance is attributed to the specific adsorption/solvation that the ions adopt at the interface with a strongly metallic Au electrode. In agreement, another recent theoretical study on Au nanoparticles has found that specific ion adsorption provides significant effects on the induced polarization of the polarizable nanoparticle surface,^[30]^ which has substantial effects on the electrostatic surface potentials and work function^[31]^ and, thus, on the EDL capacitance.

Moreover, an additional process likely contributes to the high discharging currents measured for impacting nanoparticles, i.e, the desorption of chemisorbed water molecules (which would require large developments to be included in the classical MD). Considering that PtNP collision events observed in the low potential region (Figure 3, 4) give rise to cathodic (negative) spikes, it can be concluded that the colloidal PtNPs in solution are at positive potentials compared to their potential of zero charge (PZC).^[32]^ According to experimental and computational studies of platinum single crystal surfaces, adlayer water molecules are expected to be chemisorbed to positively charged surfaces, while they desorb at potentials negative of the PZC.[^33^, ^34^] Hence, the surface of the colloidal PtNPs in solution is likely covered by chemisorbed water molecules.[^34^, ^35^] When these NPs hit the potentiostated electrode, the electrical double‐layer is quickly discharged. This refers to a change of electron density on the metal surface and respective structural reorganization at the solution side of the interface.^[36]^ Due to the applied potential being negative of the PZC, this may induce desorption of chemisorbed water molecules. As a consequence, the work function of the metal surface increases, which corresponds to an increase of its PZC.^[37]^ Thus, additional current flows to adjust the surface charge of an impacting PtNP as coverage with chemisorbed water molecules is reduced. This is similar to current flowing when surface reconstruction occurs, where a surface structure transitions to another one with a different work function.^[38]^

**Figure 7:**
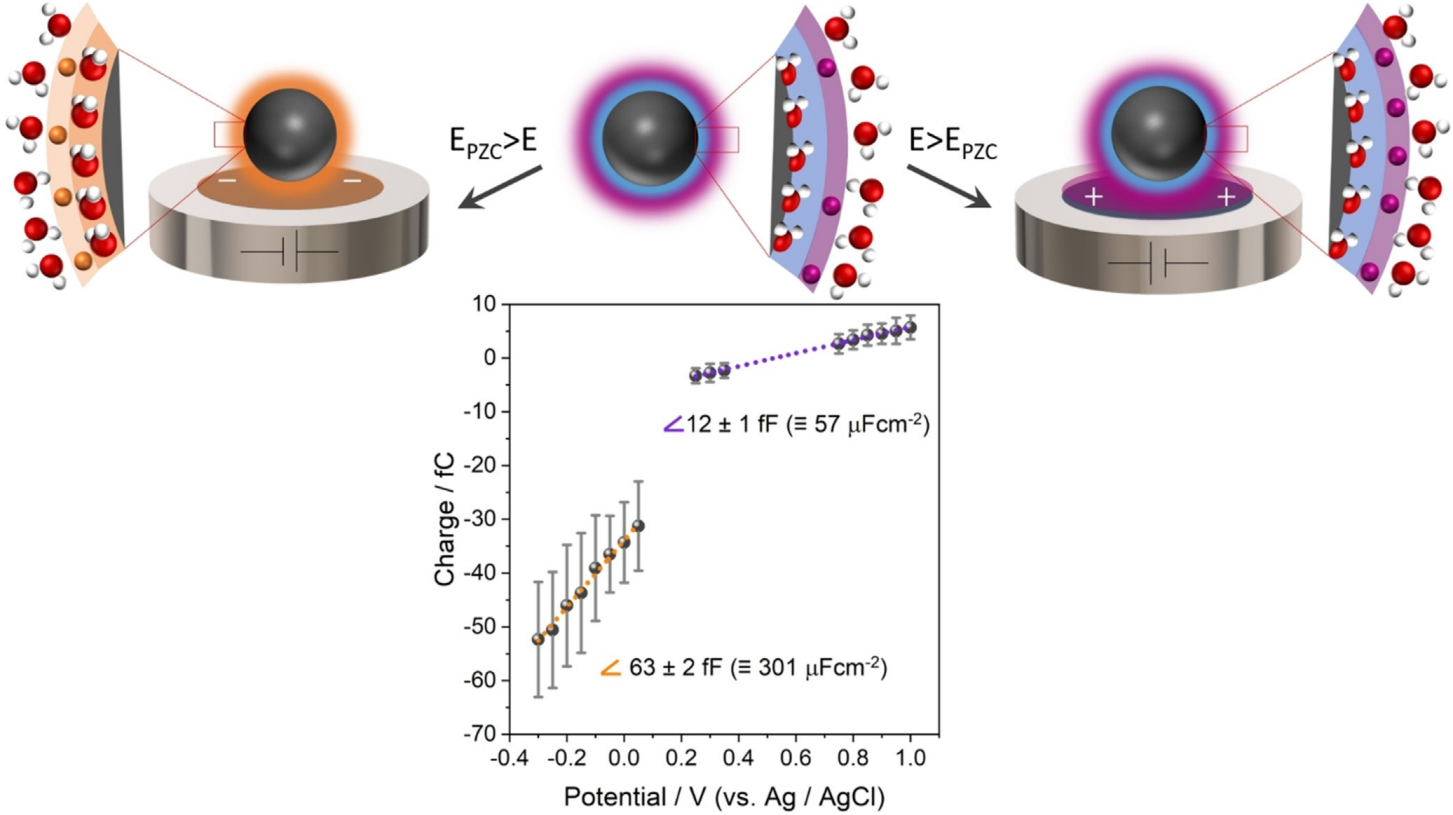
Representation for reconfiguration of NPs’ EDL (ion accumulation, water desorption) at (left) negative and (right) positive potentials vs. PZC, and related mean transferred charge vs. applied potential during 30 nm PtNP impacts in 5 mM citrate buffer saturated with Ar; error bars show the standard deviation. Red balls represent oxygen atoms, white balls hydrogen atoms, orange balls cations and purple balls anions.

For experimental assessment, impacts of PtNPs were investigated in a potential range positive of the PZC, where desorption of chemisorbed water is not expected, +0.20 V to +1.00 V vs. Ag/AgCl (+0.60 V to +1.40 V vs. RHE). As it can be seen in Figure 7, these conditions result in a capacitance value of about 60 μF cm^−2^. This is 5 times lower than that for the potential range of −0.30 V to +0.05 V vs. Ag/AgCl (+0.10 V to +0.45 V vs. RHE). For more positive potentials, the chemisorbed water does not desorb and only EDL (dis)charging causes the flow of current. Investigation of the impacts of AuNPs in a more positive potential range did not give rise to any spikes exceeding the noise level due to the lower capacitance of AuNPs in this range. From the potential value at which zero charge is transferred between impacting NPs and electrode, the potential of colloidal PtNPs in solution is estimated to be about +0.5 V vs. Ag/AgCl (+0.9 V vs. RHE).

Therefore, based on nano‐impact experiments and MD simulations, the unexpectedly high capacitance of colloidal PtNPs and AuNPs originates from two contributions: (1) preferred ion solvation near the electrode surface; and (2) desorption of chemisorbed water. The degree of commensurability between metal atom distances and distances of water‐water hydrogen bonds within the adlayer modulates the balance of metal‐water and water‐water interactions and thus determines the strength of the water adlayer binding to the metal surface. This, in turn, dictates the local hydrophobicity of the water adlayer towards the adjacent air/water‐like layer; and therefore, the probability to solvate and accumulate ions in the Helmholtz layer. Also, the contribution of water desorption depends on the chemisorptive interaction of water molecules and the metal surface. Therefore, a larger capacitance is measured for platinum than for gold nanoparticles as the water molecules bind more strongly to Pt than to Au surfaces. While contributions of the nanoscale dimension or the capping agent of the used metal particles to the high measured capacitances cannot be excluded, they are not evidently indicated by our experimental data.

## Conclusion

To improve our understanding of solid/liquid interfaces at the molecular level, we studied platinum and gold nanoparticle/water interfaces using nano‐impact electrochemistry. This technique is shown to be a new and powerful tool to gain physico‐chemical information about structural effects on the EDL capacitance of nanomaterials, without possible artifacts due to film porosity or additives. Further, it opens new possibilities to characterize colloidal NPs, as shown here by estimating their potential in solution. The charge storage ability of the double‐layer is found to increase by about one order of magnitude with respect to the predictions based on traditional mean‐field models. The large capacitance measured for platinum and gold surfaces is proposed to arise from strong interactions between the metal surface and the water adlayer. These promote water chemisorption and ion accumulation at the interface. The lower capacitance value measured for gold is ascribed to weaker binding of the water adlayer to gold vs. platinum surfaces, the latter being quantified by solvent density fluctuations analysis from classical MD simulations. Based on our findings, we propose that actively tuning solid/solvent and solvent‐solvent interactions formed by the water adlayer may emerge as a promising approach for the development of improved sustainable energy conversion and storage technologies.

## Conflict of interest

The authors declare no conflict of interest.

## Supporting information

As a service to our authors and readers, this journal provides supporting information supplied by the authors. Such materials are peer reviewed and may be re‐organized for online delivery, but are not copy‐edited or typeset. Technical support issues arising from supporting information (other than missing files) should be addressed to the authors.

Supporting Information
