# Supplementary material for: Unexpectedly High Capacitance of the Metal Nanoparticle/Water Interface: Molecular‐Level Insights into the Electrical Double Layer
Source: Angew Chem Int Ed Engl. 2021 Dec 17;61(5):e202112679. doi: 10.1002/anie.202112679 (PMC9300121; doi:10.1002/anie.202112679)
Supplement: Supplementary file 1 — Supporting Information [file ANIE-61-0-s001.pdf]

Angewandte Chemie
Eine Zeitschrift der Gesellschaft Deutscher Chemiker

Supporting Information

# Unexpectedly High Capacitance of the Metal Nanoparticle/Water Interface: Molecular-Level Insights into the Electrical Double Layer

*Mahnaz Azimzadeh Sani, Nicholas G. Pavlopoulos, Simone Pezzotti, Alessandra Serva, Paolo Cignoni, Julia Linnemann, Mathieu Salanne, Marie-Pierre Gaigeot, and Kristina Tschulik**

anie_202112679_sm_miscellaneous_information.pdf

## Table of Contents

**An overview of electrical double-layer components** ........ 2
**Experimental procedures** ........
Electrochemical measurements ........ 6
Synthesis of PtNCs ........ 7
Synthesis of AuNPs ........ 8
**Molecular Dynamics (MD) Simulations** ........ 9
**Figure S1.** Schematic representation of the electrode/electrolyte interface at negative applied potentials ........ 4
**Figure S2.** Schematic representation of the electrode/electrolyte interface at positive applied potentials ........ 5
**Results and Discussion** ........
**Figure S3.** Cyclic voltammogram of 30 nm PtNPs dropcast on a glassy carbon ........ 9
**Figure S4.** Step potential chronoamperograms recorded at different electrodes ........ 10
**Extracting information from the slope of charge-potential plots** ........ 10
**Figure S5.** TEM image of commercial PtNPs and the structure of their capping agent ........ 11
**Figure S6.** TEM image of PtNCs and the structure of their capping agent ........ 11
**Figure S7.** Compositional characterization of the as-synthesized PtNCs ........ 11
**Calculation of nanoparticles' surface area** ........ 12
**Figure S8.** Step potential chronoamperograms recorded in the presence of different nanoparticles ........ 13
**Figure S9.** TEM images of AuNPs and the structure of their capping agent ........ 13
**Figure S10.** The water density profile at the simulated Au(100)/water interface ........ 13
**Figure S11.** DLS measurement on 30 nm PtNPs ........ 14
**References** ........ 14

## An overview of electrical double-layer components

When two conducting phases, such as an electrode and an electrolyte, come into contact, their fermi levels equilibrate and a potential is set up across the interface. Depending on this potential and the composition of the solution, one phase becomes negatively charged and the other one becomes positively charged and an electrical double-layer is developed at the electrode-solution interface. In the first model proposed by Helmholtz for the structure of the electrical double-layer, it is considered that counter-charges in the solution locate at a molecular order distance from excess charges on the electrode surface.[1] Such a structure resembles a parallel-plate capacitor, whose capacitance is described by:

$$C_{\mathrm{EDL}} = \frac{\varepsilon\varepsilon_0 A}{d}$$

where $\varepsilon$ is the dielectric constant of the medium, $\varepsilon_0$ is the permittivity of free space, $A$ is the surface area of the electrode, and $d$ is the distance between the two sheets of charges. As apparent by this equation, the Helmholtz model predicts a constant capacitance for the double-layer, however, experimental results show that it changes with potential and concentration. Accordingly, either $\varepsilon$ or $d$ or both should depend on potential and concentration.

Gouy[2] and Chapman[3] by considering the fact that in contrast to charges on the electrode, the ions in the solution are not confined at a specific location close to the electrode, suggested a diffuse layer of ions in the solution. The behavior of this layer is described by the Poisson-Boltzmann equation and its thickness is essentially determined by an interplay between the tendency of the charges on the electrode to attract or repel the ions in the solution, according to their polarity, and the tendency of thermal motions to scatter them. Adjacent to the electrode, where electrostatic forces are usually able to overcome the thermal processes, the greatest concentration of counter-ions would be found, and at greater distances as the electrostatic forces become weaker, progressively less concentrations would exist. Thus, an average distance of charge separation replaces $d$ in the capacitance expression. This average distance depends on potential and electrolyte concentration. At higher electrode potentials and/or at higher electrolyte concentrations the diffuse layer becomes more compact and, hence, the double-layer capacitance rises.[4]

This model predicts a U-shaped capacitance-potential function which resembles the observed behavior at low concentrations of non-adsorbing electrolytes and at potentials not too far from the potential of zero charge. However, experiments show a flattening at high potentials, and at high electrolyte concentrations. This discrepancy is due to considering the ions as point charges in the Gouy-Chapman model, allowing them to approach the surface arbitrarily closely. Therefore, at high potentials, the effective separation

distance between the charge carriers at the electrode and in the solution decreases continuously toward zero. In reality, however, ions have a finite size and cannot approach the surface any closer than their ionic radius and if they remain solvated, the thickness of their primary solvent shell would also be added to that radius. Accordingly, Stern[5] considered a plane of closest approach for the centers of the ions at a distance from the electrode equal to their hydrated radius, named outer Helmholtz plane (OHP), and suggested that the Poisson-Boltzmann equation is only held at distances larger than this plane location. Therefore, the double-layer capacitance is made up of two components in series: one corresponds to the capacitance of the charges held at the OHP ($C_{\mathrm{OHP}}$), and the other is the capacitance of the charges at the diffuse layer ($C_{\mathrm{diffuse}}$), as shown in Figure S1 (a) and Figure S2 (a), and its capacitance is described by:

$$\frac{1}{C_{\mathrm{EDL}}} = \frac{1}{C_{\mathrm{OHP}}} + \frac{1}{C_{\mathrm{diffuse}}}$$

Thus, the smaller of the two components governs the double-layer behavior. At high potentials or in highly concentrated electrolytes, the ions in solution become tightly compressed against the OHP, and the whole system resembles the Helmholtz model. Whereas, at low potentials or at low electrolyte concentrations, the double-layer structure approaches that of the Gouy-Chapman model. Relying on this concept, Graham[6] determined the concentration independent capacitance of the Helmholtz layer as a function of potential on a mercury electrode in a highly concentrated NaF solution as an electrolyte which does not significantly adsorb on the electrode. By knowing the constant Helmholtz capacitance ($C_{\mathrm{OHP}}$) and measuring the double-layer capacitance at lower electrolyte concentrations, he calculated the potential and concentration dependent diffuse layer capacitance value ($C_{\mathrm{diffuse}}$), This agreed with electrocapillary measurements, confirming the validity of the Gouy-Chapman-Stern (GCS) model. However, the prerequisite for Graham's experiments was the use of non-adsorbing electrolytes which could not always be hold.

In the GCS model, only long-range electrostatic effects are included as the basis for accumulating the counter-ions in the solution phase and it is considered that the charge density at any point from the electrode surface to the OHP is zero and, hence, potential profile in this layer is linear and its capacitance is independent of potential. However, in real systems this is not always true.

Esin and Markov,[7] Grahame,[8] and Devanathan[9] took into account that some ions (especially large anions) can lose their hydration shell and specifically adsorb on the electrode (physical adsorption or chemisorption), forming a layer between the electrode surface and the OHP. The locus of centers of these unhydrated ions strongly adsorbed to the electrode is called the inner Helmholtz Plane (IHP), shown in Figure S2. Therefore, as derived by Devanathan, the double-layer capacitance would be described by:

$$\frac{1}{C_{\mathrm{EDL}}} = \frac{1}{C_{\mathrm{IHP}}} + \left(\frac{1}{C_{\mathrm{OHP}}} + \frac{1}{C_{\mathrm{diffuse}}}\right)\left(1 - \frac{dq_{ads}}{dq_M}\right)$$

where $C_{\mathrm{IHP}}$ and $C_{\mathrm{OHP}}$ are the capacities of the space between the electrode and the IHP and between the IHP and OHP, $C_{\mathrm{diffuse}}$ is the capacitance of the diffuse layer, and $dq_{ads}/dq_M$ represents the rate of change of the specifically adsorbed charge with charge on the metal, respectively.

In the models discussed above, the structure of the double-layer was described only based on the interfacial charge characteristics of the electrode and of the ionic species in the electrolyte. However, polar solvents like water also contribute to the potential drop across the electrode/electrolyte interface. Hence both, solvent molecules and electrode materials affect the double-layer structure and capacitance, in line with the observations made in our present study.

Accordingly, Bockris, Devanathan, and Muller suggested a model for the structure of the double-layer in which there is a strongly held and oriented layer of water molecules attached to the electrode due to the strong interaction between the charged electrode and water dipoles.[10] The complete orientation of water molecules leads to a lower dielectric constant for this layer. However, besides the full polarization of the dielectric under the strong interfacial field in the inner layer, theoretical simulations show that the strong interaction between the electrode and adsorbed water molecules disturbs their hydrogen bonding with the next water layer.[11] This in turn can lead to ion accumulation in close vicinity of the electrode, beyond the classically considered electrostatic and thermal forces mentioned above, as shown in Figure S1 (b) and Figure S2 (b).

Along with the static double-layer capacitance discussed above, faradaic pseudocapacitance can also form by a very fast reversible faradaic electron transfer between an adsorbate and electrode, such as hydrogen underpotential deposition (H-UPD) and surface oxide formation/reduction. The double-layer capacitance and pseudocapacitance behave like two capacitors in parallel,[12] thus, the overall interfacial capacitance would be the sum of these two:

$$C_{\mathrm{interface}} = C_{\mathrm{EDL}} + C_{\mathrm{pseudo}}$$

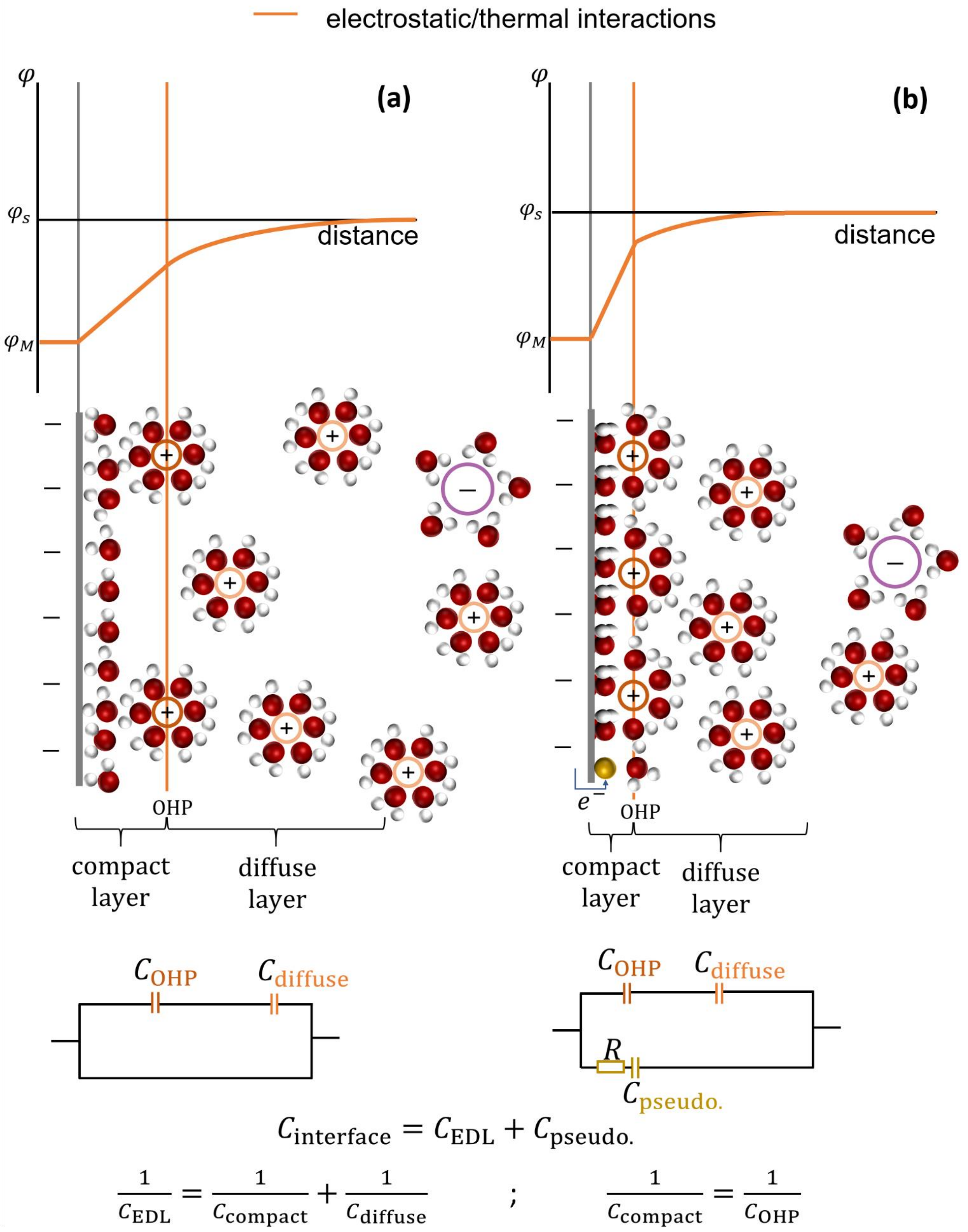


**Figure S1.** Schematic representation of the electrode/electrolyte interface at negative applied potentials, the potential profile over distance from the electrode, and the equivalent circuit, based on (a) the traditional mean-field models, (b) the modern considerations supported by our experimental findings including strongly adsorbed water molecules at the electrode surface (in red-white) which lead to higher accumulation of ions in close vicinity of the electrode, thus, a shorter Debye length. The yellow ball represents electroactive adsorbates forming the pseudocapacitance. Counter ions accumulated at the OHP are shown in dark orange and ions in the diffuse layer in light orange.

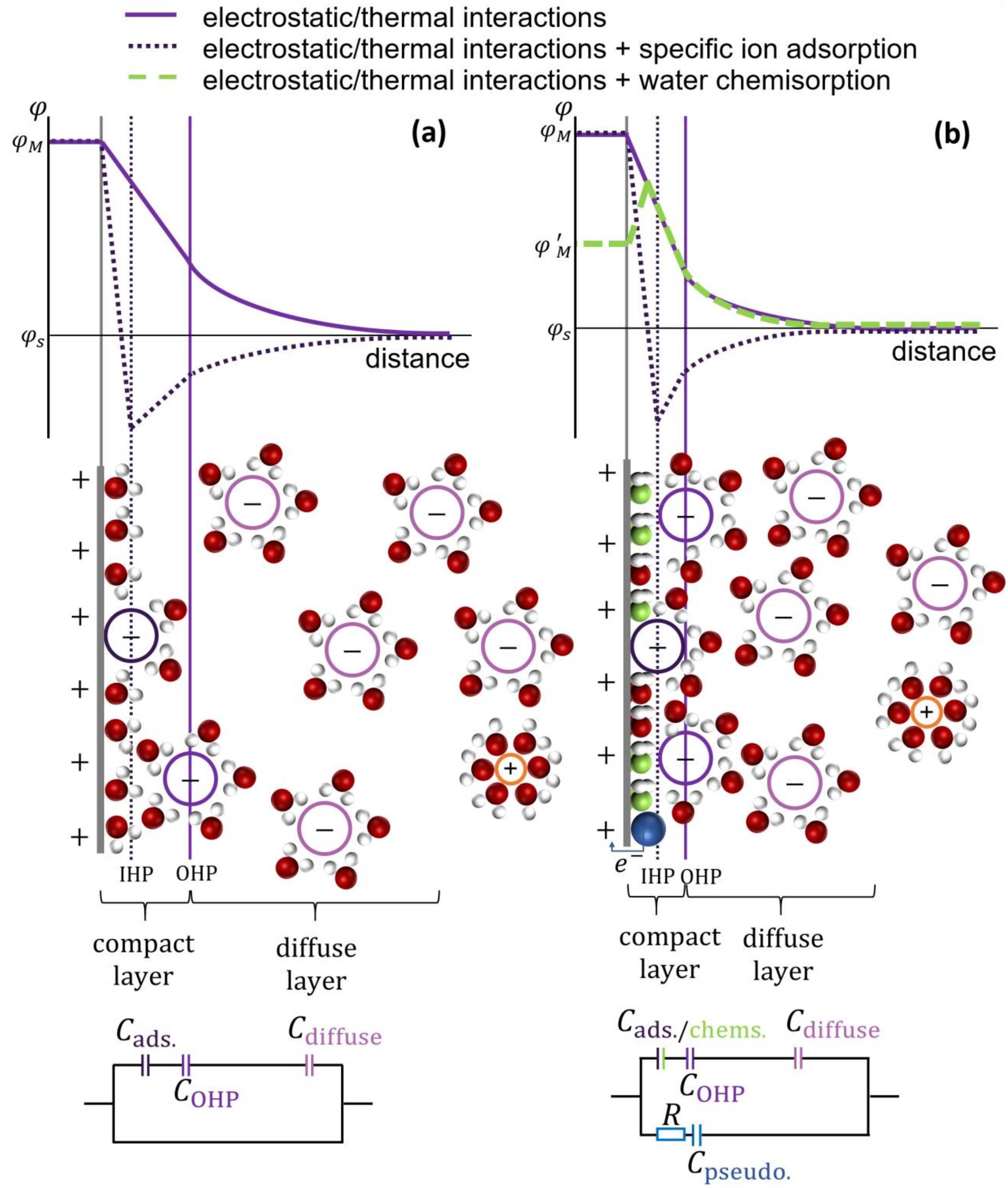


$$C_{interface} = C_{EDL} + C_{pseudo.}$$

$$\frac{1}{C_{EDL}} = \frac{1}{C_{compact}} + \frac{1}{C_{diffuse}} \quad ; \quad \frac{1}{C_{compact}} = \frac{1}{C_{ads./chems.}} + \frac{1}{C_{OHP}}$$

**Figure S2.** Schematic representation of the electrode/electrolyte interface at positive applied potentials, the potential profile over distance from the electrode, and the equivalent circuit, based on (a) the traditional mean-field models, (b) the modern considerations supported by our results including strongly adsorbed water molecules at the electrode surface (in red-white) which lead to higher accumulation of ions in close vicinity of the electrode, thus, a shorter Debye length, and chemisorbed water molecules (in green-white). The blue ball represent electroactive adsorbates forming the pseudocapacitance. Specifically adsorbed desolvated anions are shown in dark purple, counter ions accumulated at the OHP in medium purple, and ions in the diffuse layer in light purple.

In the present study, suspended nanoparticles in the electrolyte colliding with a potentiostated electrode, establish an electrical connection and equilibrate their potential with it. The nanoparticle/electrolyte interface structure and composition before impacting is different with that after the collision due to the shift of the nanoparticle potential (potential difference between the phases). The interface includes specifically adsorbed capping ligands on some of the surface sites of the nanoparticle, strongly adsorbed water molecules and counter ions in the inner layer, counter ions accumulated between the adlayer-water and the next layer of water molecules, and ions in the diffuse layer. Also, at potentials positive of PZC chemisorbed water molecules contribute to the interface composition, and desorb at negative potentials leading to an increase of the metal (nanoparticle) work function and consequently to electron transfer for maintaining the electrode potential. Therefore, the charge transfer during water chemisorption/desorption is different from pseudocapacitive behavior, where one electron per charge unit is transferred between the adsorbate and electrode.

The charge measured in capacitive nano-impact experiments is the transferred charge due to the rearrangement of the interface upon the particle electrode collision, i.e., the difference of charge stored at the interface before and after impacting. In the applied potential range, this rearrangement could include ion accumulation/depletion, water chemisorption/desorption, and – in principle – also pseudocapacitive phenomena such as: (i) desorption of the capping ligands, (ii) H-UPD, and (iii) surface-oxide reduction. We will reason below why we can exclude any of these three pseudocapacitive contributions as the cause of the detected high capacitance.

(i) Upon particle collision at the electrode, depending on the applied potential, capping ligands may be desorbed. However, no significant difference is observed in the charge-potential behavior of citrate capped Pt nanoparticles and 3-mercaptopropionic acid capped Pt nanocubes (see main text Fig. 4). Thus, we conclude that the nature of capping ligand does not influence the quantity of transferred charges in this study. Moreover, no deflection was observed in the charge-potential linear plot in the negative potential range, where desorption of the capping ligands could be expected. Hence, it can be assumed that either no desorption of the capping agent occurs or if desorption of the capping species takes place, it occurs across the full potential range tested. In the latter case, ligand desorption would add a constant contribution to each of the measured charges at all applied potentials and, hence, would not affect the slope (that is the obtained capacitance), but would only shift the charge-potential line along the y-axis.

(ii) H-UPD might take place on Pt at potentials of 0.35 V vs RHE and below, that is below −0.05 V vs Ag/AgCl, 3 M KCl RE at pH 3.2.[13] Provided that the slope of the impact charge vs potential plot showed no deflection from linear behavior in the full potential range tested (+0.05 V to −0.3 V vs Ag/AgCl, 3 M KCl RE, see Figs. 3 and 4 in main text), it can exclude H-UPD as the origin of the high measured capacitance. (We can only speculate on the reason of this absence of H-UPD, which might be attributed to the presence of the capping ligands, in line with the fact that we did also not detect H-UPD at ensembles of surface immobilized Pt nanoparticles, see Figure S3).

(iii) As inferred from the potential of suspended nanoparticles in the solution, determined by the extrapolation of the charge-potential linear plots to the potential at which no charge transfer occurs upon impacts, and as confirmed by XPS, surface of the used PtNPs is partially covered by surface oxides. These oxides can be reduced upon impact of the nanoparticle at the electrode held at a reductive potential. Since the potential window of our nano-impact analyses is more negative than the potential required for platinum oxide reduction (ca. 0.8. V vs RHE),[14] the contribution of transferred charge due to this process would be constant for each measured impact charge transferred at the different potentials; therefore, it does not affect the slope of the charge-potential plot and the capacitance derived from this.

Accordingly, the slope obtained by linear-fitting of the measured charge vs applied potential represents the static double-layer capacitance of impacting nanoparticles, raising from two contributions: ion accumulation at the interface and desorption of the chemisorbed water at negative potentials.

By looking at the density fluctuations at the metal-water interface in the absence of ions, classical MD simulations predict the way these fluctuations can promote ion adsorption through decreasing the free energy cost to form a cavity that can accommodate ions at the interface. The process of solvating an ion (or any other solute) may be decomposed in two steps: (1) creating a cavity of the right size to accommodate the ion in the liquid, accompanied by a free energy cost of cavity formation that depends on the water structure and not on the nature of the ion; (2) filling the cavity with an ion, accompanied by a free energy gain (otherwise the ion would not be soluble) due to the attractive interactions the ion makes with water that depend on the ion nature. Using this framework, it has been shown that the water structure in the adlayer can promote ion accumulation by lowering the free energy cost of step (1) compared to that in the bulk water, since creating cavities is easier at the interface than in the bulk.
From constant-potential classical MD simulations theoretical differential capacitance values are calculated from the fluctuations of the total charge on the electrode surface, using the fluctuation-dissipation relation introduced in Refs.[15] Such capacitance values incorporate the effect of ion adsorption at the interface as well as of the interfacial water network. A recent work has for example shown that specific ion adsorption at gold-water interfaces can induce very large differential capacitance values, on the order of 100 μF/cm$^2$.[16] However, common classical MD simulations do not include effects like water chemisorption, which can also contribute to large differential capacitance values, as shown by recent ab-initio MD simulations of Pt/water interfaces[15] and supported by our experimental findings.

## Experimental Procedures

### Electrochemical measurements

All electrochemical measurements were performed in a three-electrode configuration comprising a Ag/AgCl, 3 M KCl reference electrode and a platinum counter electrode, placed inside a double Faraday cage to minimize electronic noise. The reference electrode was equipped with a double junction filled with the used electrolyte solution to prevent chloride contamination of the electrolyte. The solution in the double junction was refreshed after each set of experiments.

In this work, 5 mM sodium citrate buffer with pH 3.2 (prepared by mixing tri-sodium citrate dehydrate (99.5%) and citric acid monohydrate (100%) supplied by VWR Chemicals Co., USA) was used as the electrolyte. All solutions were prepared with Millipore water (Thermo Scientific Barnstead Gen-Pure xCAD Plus, 0.055 µS cm$^{-1}$ at 25 ºC). Prior to each set of experiments the electrolyte was deaerated by purging Ar for 20 min. Nano-impact studies were performed by chronoamperometry in the presence of citrate-capped, raspberry-like PtNPs (Nanoxact, 0.05 mg ml$^{-1}$ platinum in 2 mM sodium citrate, purchased from NanoComposix Inc., USA) of ca. 30 (30 ± 3) nm and 50 (46 ± 5) nm in diameter, 3-mercaptopropionic acid-capped cubic PtNPs of ca. 18±1 nm edge length, and ascorbate-capped, spherical AuNPs of ca. 46±5 nm in dimeter.

For nano-impact experiments, as a working electrode either a carbon fiber ultramicroelectrode (Ø = 7 µm), a platinum ultramicroelectrode (Ø = 10 µm), or a gold ultramicroelectrode (Ø = 12.5 µm) was employed. Before use, they were freshly polished with 1.0 µm, 0.3 µm and 0.05 µm $Al_2O_3$ slurry and thoroughly rinsed.

Chronoamperometric nano-impact measurements were done by a potentiostat (VA-10X, NPI Electronics GmbH), equipped with a three-electrode pre-amplifier and connected to a personal computer through high-speed DA/AD data acquisition cards (DA card: USB-3101FS, AD card: USB1608FS-Plus, Measurement Computing Corp). To filter the electronic noise without altering the spike charge, a 1 kHz eight-pole low-pass Bessel filter was applied.[16] Electrochemical measurements were sampled at a data acquisition rate of 10 kHz. Multistep chronoamperograms were obtained by increasing the potential sequentially in 50 mV steps and holding for 20 s at each value. These showed well-separated transient current features, herein referred to as “spikes”, in the presence of nanoparticles. The individual charge associated to each of those spikes was determined using SignalCounter software (provided by Dr D. Omanovic, Ruder Boscovic Institute Zagreb, Croatia). The mean charge and standard deviation were determined by considering at least 50 impacts for each applied potential.

The total measurement time was limited to less than 10 minutes for each set of experiments. During this time period, no agglomeration of particles was detected in the used electrolyte, as confirmed by Dynamic Light Scattering measurements conducted by a Wyatt DynaPro NanoStar (Wyatt Technology, USA) instrument.

To determine the suitable potential window for PtNPs nano-impacts, 5 µl of 30 nm PtNPs was dropcast on a freshly cleaned glassy carbon electrode, GCE, (Ø =25 mm) polished with 1.0 µm, 0.3 µm and 0.05 µm $Al_2O_3$ slurry and thoroughly rinsed with ultrapure water, and dried under a mild Ar stream. This was used as the working electrode to perform cyclic voltammetry. The experiment was done in 5 mM deaerated sodium citrate buffer of pH 3.2 as the electrolyte. Cyclic voltammetry was performed with a PalmSens 3 (PalmSens BV, Netherlands) potentiostat from +0.60 V to −0.30 V and from +0.60 V to −0.40 V vs. Ag/AgCl (+1.00 V to +0.10 V and +1.00 V to 0.00 V vs. RHE) with a scan rate of 0.025 Vs$^{-1}$.

**Synthesis of PtNCs**

***Materials.*** All chemicals were used as purchased unless otherwise specified. Cyclohexanone (99 %), dodecylamine (99 %), 3-mercaptopropionic acid (99 %), octylamine (99.5 %), oleic acid (99 %), platinum acetylacetonate (99.98 %), sulfuric acid (95 %), tetramethylammomium hydroxide pentahydrate (95 %) were purchased from Merck. Ethanol (90 %) and diphenyl ether (99 %) were commercially available from Alfa Aesar and used as received. All heating steps described in the following were performed using silicon oil baths on IKA C-MAG HS 7 magnetic stirred/hotplates. All centrifugation was performed in 15 mL centrifuge tubes (unless otherwise stated) using a rotor with a radius of 11 cm.

***General Strategy for PtNC Synthesis***. The synthesis of single crystalline Pt-nanoparticles with cubic crystal habit was based on the GRAILS (gas reducing agent in liquid solution) method of Yang and coworkers, with minor modifications.[19] Details of the method used herein are included below.

***Preparation of Hot Injection stock solution.*** $Pt(acac)_2$ (0.200 g, 5.09 x 10$^{-4}$ moles), dodecylamine (10.0 mL, 8.06 g, 4.34 x 10$^{-2}$ moles), and oleic acid (0.5 mL, 0.448 g, 1.58 x 10$^{-3}$ moles) were added into a 20 mL scintillation vial equipped with a 10 mm Teflon coated stir bar in an $N_2$ filled glovebox. The vial was sealed with a septum-screwcap, and vortex mixed until homogeneous (2 minutes). Subsequently, the vial was heated to 80 ºC in a thermostated oil bath at 200 RPM for 30 minutes under vacuum to remove any adventitious moisture/oxygen, before being backfilled with Ar using standard Schlenk line technique. Before injection to the reaction solution, the hot injection stock was heated to 150 ºC, to control the size of Pt-nanocubes by modification of nucleation rate as a function of reaction temperature.

***Preparation of Carbon Monoxide Gas.*** Carbon monoxide gas was produced on demand by the dropwise addition of formic acid to sulfuric acid at 50 ºC. This method was preferred over the use of a carbon monoxide gas cylinder, as the gas-phase reducing agent could be produced on demand in small quantities, with rate tunable by formic acid addition rate and sulfuric acid temperature. *We emphasize the importance of performing all CO preparations in a well-ventilated hood in the presence of a carbon monoxide detector. Additionally, before CO production is initiated, one should ensure both the CO production vessel and the reaction vessel (to which CO will be transferred by cannula) are under positive Ar-flow using standard Schlenk line technique. In such a manner, all unreacted CO gas is directed into the Schlenk line and subsequently vented into a well-ventilated chemical hood.*

For this preparation, concentrated sulfuric acid (10 mL) was added to a 50 mL three neck round bottom flask equipped with a reflux condenser, ½” Teflon coated stir bar, and rubber septa. The contents of the flask were then heated to 50 ºC in a thermostated oil bath at 300 RPM under Ar flow using standard Schlenk line technique. Before CO gas production, the flask was equipped with a vent needle connected to a drying tube filled with activated 3 Å molecular sieves, to remove any adventitious moisture produced in the formic acid

dehydration reaction. To initiate the production of CO gas, concentrated formic acid was added dropwise to the sulfuric acid solution at a rate of 50 µL/min, which resulted in immediate mild bubbling in the reaction flask, indicative of production of CO.

***General synthesis of PtNCs.*** Dodecylamine (5.0 mL, 4.03 g, 2.17 x $10^{-2}$ moles) and diphenyl ether (0.500 mL, 0.484 g, 2.84 x $10^{-3}$ moles) were added to a 15 mL three neck round bottom flask equipped with a reflux condenser, ½" Teflon coated stir bar, and rubber septum. The contents of the vial were heated to 80 ºC under vacuum for 30 minutes to remove any adventitious moisture/oxygen, followed by backfilling with Ar using standard Schlenk line technique. The contents of the vial were then heated to 210 ºC in a thermostated oil bath at 300 RPM. Once at 210 ºC, the reaction solution was bubbled with CO gas for 15 minutes before the hot injection stock solution (5.00 mL) at 150 ºC was injected via a glass syringe to the reaction flask at 210 ºC. The reaction was allowed to proceed for 30 minutes after injection under continual CO-flow before being quenched by removal from the oil bath. At this point, CO-production was ceased by stopping the formic acid addition and removing the CO-production flask from the oil bath.

***Post synthesis.*** A single workup cycle was employed to remove excess diphenylether, dodecylamine, oleic acid, and Pt-precursor from the synthesized nanoparticles. For this, the crude reaction mixture was diluted with toluene (5.0 mL) and added to a 15 mL centrifuge tube, followed by addition of ethanol (5.0 mL) to precipitate the nanocrystals. Subsequently, the dispersion was centrifuged at 1250 RPM for 12 minutes to result in a clear/colorless supernatant and a light grey and distributed pellet consisting of the PtNCs. The supernatant was discarded, and the pellet was immediately carried through to ligand exchange as described below.

***Ligand Exchange of PtNCs.*** The method used herein was developed based on previously reported FePt ligand exchanges, which take advantage of the sulfophilic nature of Pt-surfaces to enable exchange of native carboxylate and amine ligands for water-dispersable alkylthiols.[20] Cyclohexanone was chosen as the solvent for the ligand exchange as it both dissolves 3-MPA and disperses the as synthesized PtNPs. First, a solution of octylamine (0.5 mL, 0.391 g, 3.03 x $10^{-3}$ moles) in n-hexane (9.50 mL) was prepared (0.303 M octylamine), and the purified nanocrystal pellet was dispersed in this hexane/octylamine stock solution (2.5 mL) via sonication (10 minutes) and subsequent vortex mixing. The octylamine was added to prevent aggregation of the PtNPs upon re-dispersion. Next, in a 4 dram scintillation vial equipped with a 10 mm Teflon coated stir bar, 3-MPA (0.500 mL, 0.609 g, 5.72 x $10^{-2}$ moles) was added followed by cyclohexanone (0.5 mL), resulting in a 5.72 M 3-MPA ligand exchange stock solution. Next, a portion of the PtNP in hexane/octylamine was pipetted on top of the MPA/cyclohexanone layer (0.25 mL), resulting in a biphasic system. The dispersions were allowed to stir at 50 RPM at room temperature for 30 minutes, by which point a homogeneous grey dispersion was observed to have formed. The dispersion was then transferred to a 15 mL centrifuge tube, followed by addition of ethanol (1.0 mL) to precipitate the PtNPs. Subsequently, the dispersion was centrifuged at 1250 RPM for 12 minutes, resulting in a clear/colorless supernatant and a light grey and distributed pellet. Next, to remove excess MPA from the PtNPs, the pellets were washed with ethanol (2.0 mL). Brief sonication resulted in a slightly cloudy grey dispersion, which was then centrifuged at 1250 RPM for 12 minutes to result in a clear/colorless supernatant and a light grey pellet. The supernatant was then discarded. Subsequently, a solution of TMAH•5$H_2O$ (0.500 g, 2.76 x $10^{-3}$ moles) in ethanol (10 mL) was prepared (275 mM TMAH•5$H_2O$), and the PtNPs were dispersed in the TMAH stock solution (2.0 mL) via sonication (10 minutes). The samples were then precipitated via centrifugation at 2500 RPM for 12 minutes, resulting in a grey/brown pellet and a clear/colorless supernatant. The supernatants were discarded, and the pellets were dried in vacuum for 10 minutes to remove residual ethanol, yielding ~ 2-4 mg of ligand exchanged Pt nanocubes. Next, DI-$H_2O$ (1.0 mL) was added to the pellets, resulting in immediately a clear deep brown dispersion free of any particulates. The dispersion was then passed through a 0.25 microneter syringe filter, and used as an aqueous solution for nano-impact experiments.

**Synthesis of AuNPs**

***Materials.*** All chemicals were used as purchased unless otherwise specified. Tetrachloroauric acid (99.99 % metal basis) and silver nitrate (99.9995 % metal basis) were purchased from Alfa Aesar.

The solutions of the metal salts were prepared freshly before the synthesis.

Sodium citrate dihydrate 99.5 % (VWR Chemicals) and L(+)-ascorbic acid ≥ 99 % (ROTH) solution were stored at −25 ºC.

The heating steps were carried out using IKA RH basic 2 magnetic stirred/hotplates equipped with custom adaptors for round bottom flask. Centrifugation was performed in 50 mL centrifuge tubes on a 5810 R, Eppendorf centrifuge.

Ultra-pure water (Thermo Scientific Barnstead Gen-Pure xCAD Plus, 0.055 µS $cm^{-1}$ at 25 ºC) was used to prepare all solutions and in each of the reaction steps.

All the glassware was cleaned overnight in a base bath (0.1 M potassium hydroxide in isopropanol), in an acid bath (0.1 M hydrochloric acid in water) and rinsed with ultra-pure water before use. Teflon coated stirring bars were cleaned in aqua regia and rinsed with ultra-pure water.

***General Strategy for AuNPs Synthesis.*** 46 ± 5 nm AuNPs were synthetized by using a modified version of the seed mediated approach optimised by Murphy and coworkers.[21] Traces of silver nitrate were added to favour the formation of spherical nanoparticles instead of nanorods. The 25 ± 4 nm Au seeds used in the procedure were synthetized by following the citrate based method originally proposed by Turkevic.[22] The detailed procedures followed in this work are described below.

***Preparation of Au seeds.*** 250 mL of ultra-pure water were added to a 500 mL three neck round bottom flask, equipped with a reflux condenser and a 30 mm Teflon coated stir bar, and heated to 100 ºC. 500 µL of a 50 mM tetrachloroauric acid trihydrate solution (2.50×$10^{-5}$ moles) and 1.25 mL of a 60 mM trisodium citrate dihydrate solution (7.50 x $10^{-5}$ moles) were added. A red suspension was formed during the first minutes after reagents addition. The suspension was heated for one additional hour before being allowed to cool down at room temperature and adding 2.5 mL of the 60 mM trisodium citrate dihydrate solution (1.5 x $10^{-4}$ moles). 5 mL of the final suspension was centrifuged at 15000 RCF for 15 minutes, 4 mL of supernatant was removed to obtain 1 mL of the concentrated seeds.

***Preparation of AuNPs.*** 30 mL of ultra-pure water, 1 mL of the concentrated Au seeds, 85 µL of a 50 mM tetrachloroauric acid trihydrate solution (4.25 x $10^{-6}$ moles) and 8.76 µL of a 10 mM silver nitrate solution (8.76 x $10^{-8}$ moles) were added to a 100 mL round bottom flask, equipped with an equilibrated dropping funnel and a 20 mm Teflon coated stirr bar, and stirred at room temperature. 86 µL of a

100 mM L(+)-ascorbic acid solution (8.6 x $10^{-6}$ moles) and 20 mL of ultra-pure water were transferred to a dropping funnel and added to the reaction mixture, dropwise (25 minutes for completing the addition). The pale red reaction mixture darkened slowly. Once terminated the reductant addition, the suspension was stirred for one hour.

***Post synthesis.*** The AuNPs suspension was centrifuged at 15000 RCF for 15 minutes. Most of the supernatant was removed and 500 µL of a concentrated AuNPs suspension was obtained.

**Characterization of the nanoparticles by TEM**

***Commercial PtNPs.*** Size and structure of the used commercial PtNPs were characterized by transmission electron microscopy (TEM) recorded on a JEOL, JEM-2800 with a Schottky type electron gun at 200 kV. Samples were prepared for imaging by dropcasting 5 µL of a stock PtNP suspension onto an ultra-thin carbon film (3 nm) on Lacey carbon film 400 mesh Cu (Plano GmbH). After dropcasting, grids were left to dry overnight before examination.

***Synthesized PtNCs.*** The size and structure of the PtNCs have been characterized by TEM obtained on a FEI Tecnai G2 T20 S-Twin TEM operated at 200 kV with a LaB6 electron source using carbon coated copper grids square, 200 mesh (Electron Microscopy Sciences, USA). High Resolution STEM imaging was performed on an FEI Titan High Resolution aberration-corrected TEM/STEM operated at 200 kV. (See Figures S6 and S7)

***Synthesized AuNPs.*** Size and structure of the AuNPs was determined by TEM microscopy carried out on a JEOL JEM-2800 microscope working with a Schottky type electron gun at 200 kV. Image were recorded on a Gatan OneViewIS camera. The point to point resolution was 0.20 nm. The suspensions were dropcast on ultra-thin carbon film (3nm) on Lacey carbon film 400 mesh Cu grids (Plano GmbH). Figure S8 depicts TEM images of the Au seeds and AuNPs.

**Molecular Dynamics (MD) Simulations**

A classical MD simulation of a liquid slab composed by 3481 water molecules between two planar Au(100) surfaces (each electrode made of 5 layers of 162 gold atoms) has been performed at a fixed potential difference of 0 V using the MetalWalls code.[23] 2D periodic boundary conditions were employed, with no periodicity on the z-direction (perpendicular to the gold surfaces), and box dimensions along x and y directions of $L_x$=$L_y$=36.63 Å. The SPC/E model[24] was chosen for water, while Lennard-Jones parameters introduced by Heinz et al.[25] for Au(100) and Lorentz-Berthelot mixing rules were used to model the interactions between all atoms. Electrostatic interactions were computed using a 2D Ewald summation method, with a cut-off of 12 Å for the short-range part of the Coulomb interactions and a cut-off of 15 Å for the intermolecular ones. The simulation box was equilibrated at constant atmospheric pressure by applying a constant pressure force to the electrodes. The electrodes separation was then fixed to the equilibrium value of 78.6 Å (for which the water density in the middle of box corresponds to the bulk value, for the rest of the equilibration (5 ns, NVT ensemble, $T$ = 298K) and for the production (100 ns, NVT, $T$= 298K). The equation of motions were solved using a timestep of 1 fs during equilibration and 2 fs during production.

The free ener$\delta\mu_v(z)$gy (shown in Figure 7 (b)) is obtained as the difference between the free energy cost to form a cavity at a distance $z$ from the adlayer formed in contact with the metal surface, $\Delta\mu_v(z)$, and in the bulk. It is calculated from the MD simulation by monitoring the probability ($P_v(0,z)$) to find zero water oxygen centers in the probing volume, V:[26]

$$P_v(0,z) = e^{-\beta\Delta\mu_v(z)}$$

Where $\beta = 1/k_B T$, with $k_B$ being the Boltzmann constant and $T$ the temperature ($T$=298K). A spherical probing volume of 3 Å radius has been adopted. The result for the water/Au(100) system is compared in Figure 7 (b) of the main text to the ones previously obtained by Limmer et al.[26] for Pt(100) and Pt(111) systems, employing a similar computational setup as in the present simulation.

## Results and Discussion

As shown in Figure S3, no hydrogen under potential deposition (H-UPD) is realized at the nanoparticles during cyclic voltammetry, although this could be expected for Pt electrodes. This may be attributable to the strong specific adsorption of citrate anions on the PtNPs as has been reported by Attard et al.[27] Therefore, H-UPD cannot interfere with the capacitance measurements.

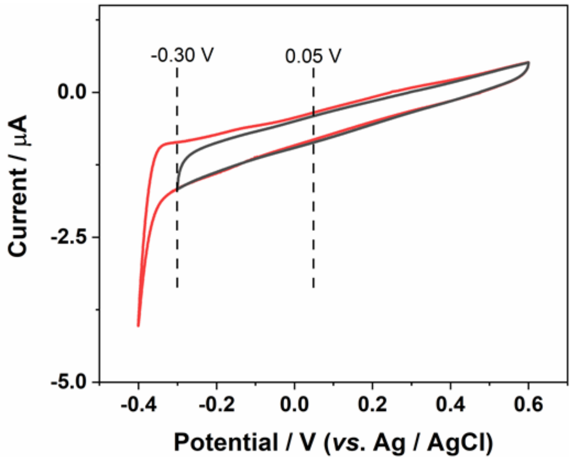


**Figure S3.** Representative cyclic voltammogram of 30 nm PtNPs dropcast on a glassy carbon electrode in 5 mM citrate buffer with pH 3.2, saturated with Ar, with a scan rate of 0.025 $Vs^{-1}$.

In order to figure out the origin of the capacitive spikes being either charging of the impacting nanoparticle or perturbation of the electrode double-layer by the nanoparticle, step potential chronoamperometry was run using different ultramicroelectrodes (carbon, Pt, Au) as the working electrode (Figure S4). During these experiments changes of the transferred charge were investigated as a function of the applied potential. Spikes from −0.30 V to +0.05 V vs. Ag/AgCl (+0.10 V to +0.45 V vs. RHE) were included in data analysis to avoid any parallel faradaic reaction and to be able to precisely resolve spikes from background noise.

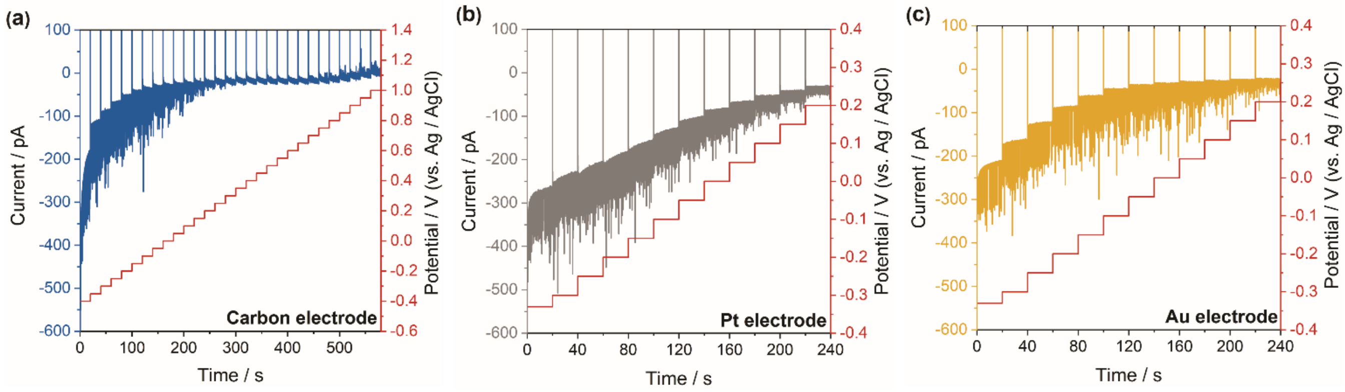


**Figure S4.** Representative step potential chronoamperograms recorded at a) carbon, b) platinum, and c) gold ultramicroelectrodes in 5 mM citrate buffer saturated with Ar are plotted in blue. The applied potentials and the associated second y-axis are shown in red.

Observed background currents are in the typical range for microelectrodes and may originate from oxygen reduction caused by very small amounts of oxygen entering the deoxygenated electrolyte upon NP injection into the measurement cell, although the NP suspension was deoxygenated by Ar purging prior to injection, as well. The current associated to this is at least 100 times smaller than expected for air saturated solution and can, hence, be concluded not to significantly alter the capacitance measurements.

### Extracting information from the slope of charge-potential plots

Upon colliding with the working electrode, the nanoparticle changes its potential ($E_{NP,S}$) to the applied potential at the working electrode ($E$).[28] Therefore, its surface charge ($q$) also changes depending on its potential change ($\Delta E$) and its capacitance ($C_{NP}$). As a result, the charge measured during a nano-impact reflects the alteration of the nanoparticle's surface charge ($\Delta q$) due to changing its potential.

$$\Delta q = C_{\mathrm{NP}} \cdot \Delta E$$

If we assume that capacitance of the nanoparticle $C_{NP}$ is constant over the considered potential range, we get:

$\Delta q_1 = C_{\mathrm{NP}} \cdot \Delta E_1$

$\Delta q_2 = C_{\mathrm{NP}} \cdot \Delta E_2$

$\Delta E_1 = E_1 - E_{\mathrm{NP,S}}$ , $\Delta E_2 = E_2 - E_{\mathrm{NP,S}}$

$$\text{slope} = \frac{\Delta q_2 - \Delta q_1}{E_2 - E_1} = \frac{(C_{\mathrm{NP}} \cdot \Delta E_2) - (C_{\mathrm{NP}} \cdot \Delta E_1)}{E_2 - E_1} = \frac{C_{\mathrm{NP}}(\Delta E_2 - \Delta E_1)}{E_2 - E_1} = \frac{C_{\mathrm{NP}}\left((E_2 - E_{\mathrm{NP,S}}) - (E_1 - E_{\mathrm{NP,S}})\right)}{E_2 - E_1} = C_{\mathrm{NP}}$$

Therefore, a constant slope of charge-potential plots represents a difference quotient, which is a reasonable estimate for the differential capacitance of the impacting nanoparticles in the considered potential range when potential steps are infinitesimally small.

In this work two sizes of commercial citrate capped- PtNPs with nominal diameters of 30 nm (30 ± 3 nm) and 50 nm (46 ± 5 nm) were used. As shown by TEM, these particles are raspberry-like clusters formed from the aggregation of smaller particles with sizes in the range of 3–5 nm.

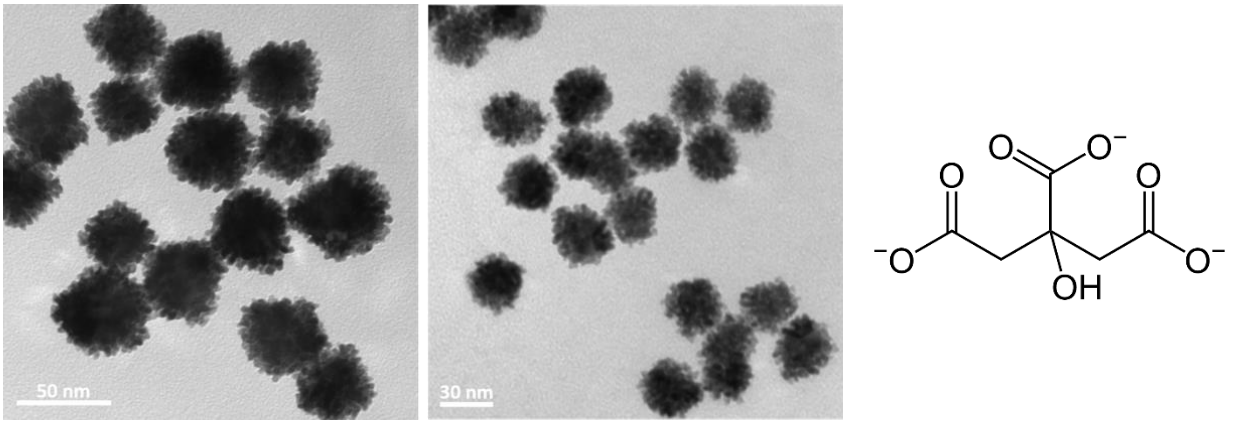


**Figure S5.** TEM images of nominally 50 nm (left) and 30 nm (right) commercial PtNPs, and their capping agent (citrate) structure.

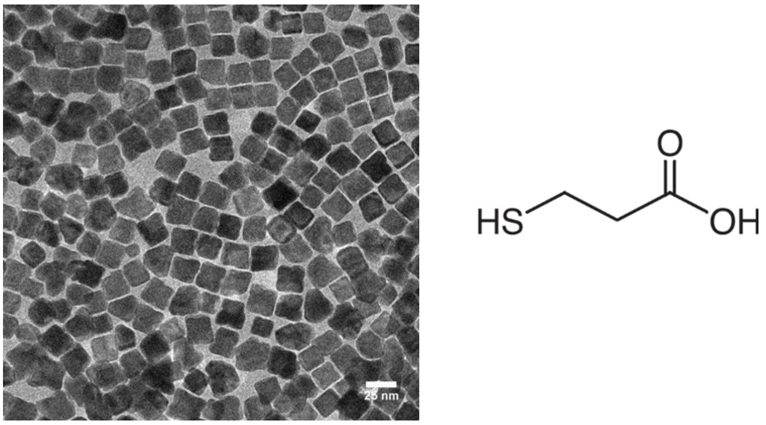


**Figure S6.** TEM image of 18 nm PtNCs afforded by hot injection at 150 ºC and 30 minutes of reaction time, and the structure of their capping agent (3-mercapto propionic acid).

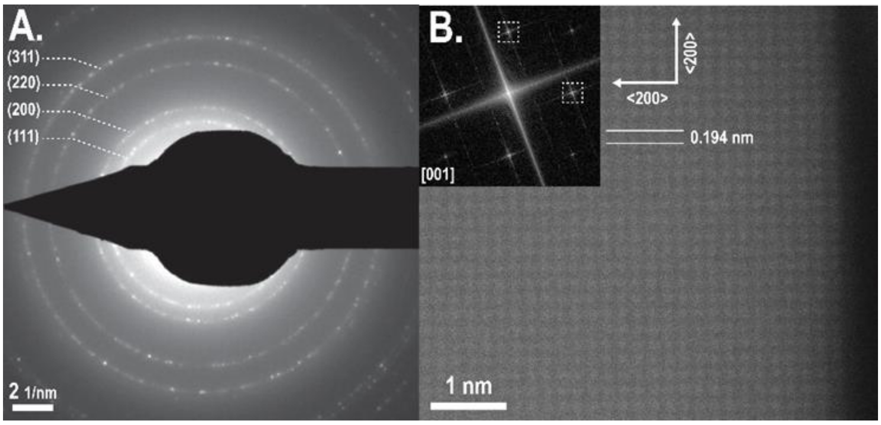


**Figure S7.** Compositional characterization of the as-synthesized PtNCs. A) SAED pattern obtained at low magnification for close-packed particles, demonstrating characteristic reflections assigned to the (111), (200), (220), and (311) crystallographic planes of face centered cubic (fcc) platinum (2.24, 1.95, 1.38, 1.18 angstrom, respectively; JCPDS No. 04-0802). B) HRSTEM image of a single Pt-NC edge recorded along the [001] zone axis with corresponding fast Fourier transform (FFT) (inset) demonstrating reflections from the (200) planes and characteristic lattice spacings of 0.194 nm).

**Calculation of nanoparticles' surface area**

TEM images show that 30 nm and 50 nm PtNPs are clusters of smaller, spherical nanoparticles, described previously as "mesoporous" nanoparticles.[29] Measuring the accessible surface of these particles based on the TEM images is not directly possible. Therefore, we estimated the area of the raspberry-like particles as the sum of the area of their constituting small particles. For a close-packing-type arrangement of these spheres in the aggregate, the fractional filling efficiency is 0.74.[30] Thus, the number of small particles in the aggregate nanoparticle would be as follows:

$$n_{\text{small NP}} \times V_{\text{small NP}} = 0.74\, V_{\text{cluster}}$$

here $V_{\text{small NP}}$ is the volume of constituting small particles and $V_{\text{cluster}}$ is the volume of clusters.

$$V_{\text{sphere}} = \frac{4}{3}\pi R^3$$

where $R$ is the radius of the sphere. Therefore:

$$\frac{V_{\text{cluster}}}{V_{\text{small NP}}} = \left(\frac{R_{\text{cluster}}}{R_{\text{small NP}}}\right)^3$$

For the 30 ± 3 nm clusters the radius of the constituting spheres $R_{\text{small NP}}$ is about 1.5 nm and for the 46 ± 5 nm clusters $R_{\text{small NP}}$ is about 2.3 nm. Therefore, each of the raspberry-type Pt nanoparticles constitutes of about 740 spheres.

$$n_{\text{small NP}} = 740$$

The maximum surface area of the aggregate ($A_{\text{cluster}}$) is the sum of small NPs surface area ($A_{\text{small NP}}$):

$$A_{\text{small NP}} = 4\pi R^2_{\text{small NP}}$$

$A_{\text{cluster}} = 740\ A_{\text{small NP}}$

Therefor their surface area can be estimated as 2.1 × 10$^{-10}$ cm$^2$ and 4.9 × 10$^{-10}$ cm$^2$, respectively.

The surface area of the used Pt nanocubes with 18 ± 1 nm edge length ($a$) is 1.9 × 10$^{-11}$ cm$^2$ calculated as follows:

$$A = 6\,a^2$$

The surface area of the used Au nanoparticles with 46 ± 5 nm diameter ($d$) is 6.6 × 10$^{-11}$ cm$^2$ calculated as follows:

$$A = 4\,\pi\left(\frac{d}{2}\right)^2$$

To elucidate the origin of high specific capacitance measured for nominally 30 nm cluster PtNPs, which can be due to porosity or curvature effects of nanoclusters or divergence from classical Gouy-Chapman-Stern (GCS) model for the EDL, we studied particles of different sizes, morphologies and materials. This was done for nominally 50 nm raspberry-like PtNPs, 18 nm PtNCs and 46 nm AuNPs using a platinum working electrode, related chronoamprograms can be seen in Figure S8.

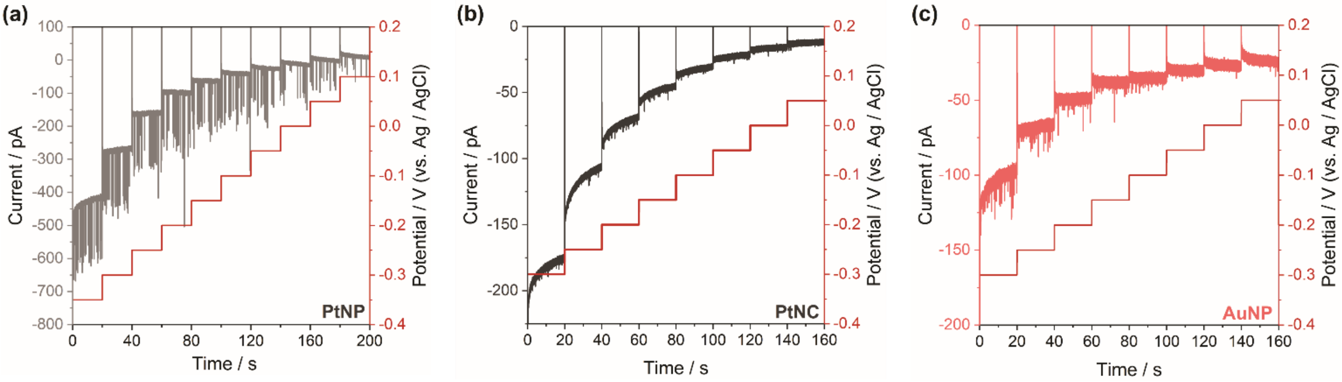


**Figure S8.** Representative step potential chronoamperograms recorded at a Pt electrode in 5 mM citrate buffer saturated with Ar in the presence of a) 50 nm raspberry-like PtNPs, b) 18 nm PtNCs, and c) 46 nm AuNPs. Applied potentials and the associated y-axes are plotted in red.

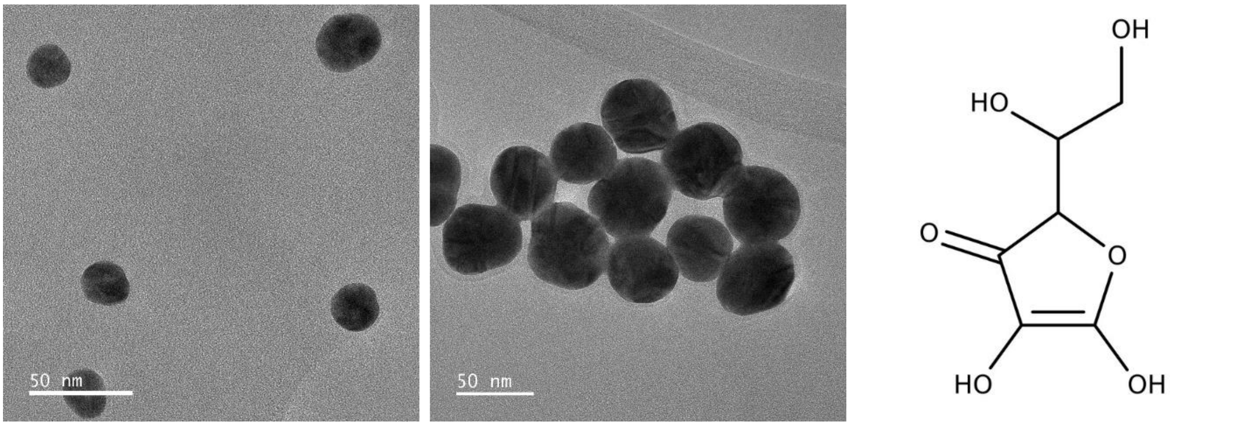


**Figure S9.** TEM images of 25 nm Au seeds (left) and 46 nm AuNPs (right), and the structure of their capping agent (ascorbic acid).

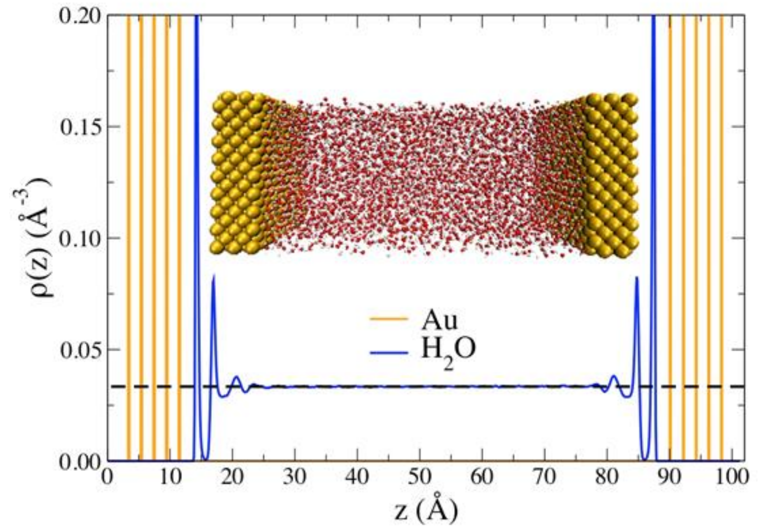


**Figure S10.** Average density profile of water molecules (blue) and Au atoms (orange) as a function of the direction normal to the Au(100) surface (z). A representative snapshot of the simulation box is also reported. The water density in the middle of the box corresponds to the bulk water density (dashed black line), ensuring that the system has been properly equilibrated.

Dynamic light scattering (DLS) measurements were conducted on 30 nm PtNPs in ultrapure water, 5 mM and 10 mM sodium citrate buffer, to ensure the stability of PtNPs in the electrolyte solution used for nano-impact studies. By comparing the 10 mM and 5 mM sodium citrate buffer curves with ultrapure water curve in Figure S11, it is concluded that during the time span of the nano-impact experiments (maximum 10 minutes), agglomeration of the particles is happening in 10 mM sodium citrate buffer, but in 5 mM solution the particles are stable against agglomeration. Accordingly, a 5 mM solution was used for the single particle capacitance measurements.

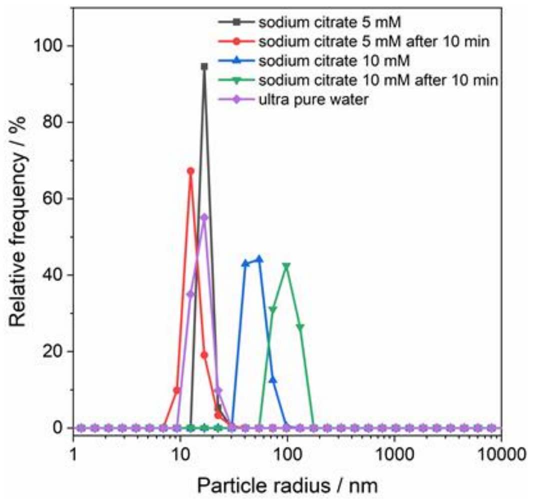


**Figure S11.** DLS measurement on 30 nm PtNPs performed in 5 mM and 10 mM sodium citrate buffer in the time span of 10 minutes and also in ultrapure water.

## References


[1] H. Helmholtz, Helmholtz's Wissenschaftliche Abhandlungen, Physik. Tech. Reichsanstalt., **1882**, *1*, 925.

[2] G. Gouy, Sur la constitution de la charge electrique a la surface d'un electrolyte, **1910**, *9*, 457.

[3] D. L. Chapman, LI. *Philosophical Magazine and Journal of Science*, **1913**, *25*, 475.

[4] A. J. Bard and L. R. Faulkner, Electrochemical methods. Fundamenta and applications, JOHN WILEY &SONS, INC., New York, United states of America, **2001**.

[5] O. Stern, *Zeitschrift für Elektrochemie und angewandte physikalische Chemie*, **1924**, *30*, 508.

[6] D. C. Grahame, *J Am. Soc.*, **1954**, *76*, 4819.

[7] O. A. Esin and B. F. Markov, *Zh. Fiz. Khim*, **1939**, *13*, 318.

[8] D. C. Grahame, *J. Electrochem. Soc.*, **1951**, *98*, 343.

[9] M. A. V. Devanathan, *Trans. Faraday Soc*., **1954**, *50*, 373.

[10] J. O. Bockris, M. A. V. Devanthan and K. Mueller, *Proc. R. Soc. Lond. A*, **1963**, *274*, 55.

[11] a) D. T. Limmer, A. P. Willard, P. Madden, D. Chandler, *PNAS* **2013**, *110*, 4200; b) H. Acharya, S. Vembanur, S. N. Jamadagni, S. Garde, *Faraday Discussions* **2010**, *146*, 353; c) A. Serva, M. Salanne, M. Havenith, S. Pezzotti, *PNAS,* **2021**, *118,* e2023867118.

[12] J. O'M Bockris and S. Srinivasan, Fuel Cells: Their Electrochemistry, McGraw-Hill Book Company, New York, **1969**.

[13] M. Łukaszewski, M. Soszko and A. Czerwiński, *Int. J. Electrochem. Sci.*, **2016**, *11*, 4442.

[14] P. P. Lopes, D. Tripkovic, P. F. Martins, D. Strmcnik, E. A. Ticianelli, V. R. Stamenkovic and N. M. Markovic, *J. Electroanal. Chem.*, **2018**, *819*, 123.

[15] a) D. T. Limmer, C. Merlet, M. Salanne, D. Chandler, P. A. Madden, R. van Roij, B. Rotenberg, *Phys. Rev. Lett*. **2013**, *111*, 106102; b) L. Scalfi, D. T. Limmer, A. Coretti, S. Bonella, P. A. Madden, M. Salanne, B. Rotenberg, *Phys. Chem. Chem. Phys*., **2020**, *22*, 10480.

[16] A. Serva, L. Scalfi, B. Rotenberg, M. Salanne, *The Journal of Chemical Physics* **2021**, *155*, 044703.

[17] J. B. Le, Q. Y. Fan, J. Q. Li, J. Cheng, *Sci. Adv*. **2020**, *6*.

[18] K. Kanokkanchana, E. N. Saw, K. Tschulik, *ChemElectroChem* **2018**, *5*, 3000–3005.

[19] W. Zhou, J. Wu, H. Yang, *Nano Letters* **2013**, *13*, 2870-2874.

[20] a) H. G. Bagaria, E. T. Ada, M. Shamsuzzoha, D. E. Nikles, D. T. Johnson, *Langmuir* **2006**, *22*, 7732–7737; b) R. M. Dragoman, *et al. Chemistry of Materials* **2017**, 29, 9416–9428.

[21] N. R. Jana, L. Gearheart, C. J. Murphy, *Chemistry of Materials* **2001**, *13*, 2313–2322.

[22] J. Turkevich, P. C. Stevenson, J. Hillier, *Discuss. Faraday Soc.* **1951**, *11*, 55– 75.

[23] A. Marin-Laflèche, *et al. Journal of Open Source Software* **2020**, *5*, 2373–2377.

[24] H. J. C. Berendsen, J. R. Grigera, T. P Straatsma, *The Journal of Physical Chemistry* **1987**, *91*, 6269–6271.

[25] H. Heinz, R. A. Vaia, B. L. Farmer, R. R. Naik, *The Journal of Physical Chemistry C* **2008**, *112*, 17281–17290.

[26] D. T. Limmer, A. P. Willard, P. Madden, D. Chandler, *PNAS* **2013**, *110*, 4200–4205.

[27] G. A. Attard, *et al*. *Journal of Electroanalytical Chemistry* **2013**, *688*, 249–256.

[28] P. Peljo, J. A. Manzanares, H. H. Girault, *Chemical Science* **2017**, *8*, 4795–4803.

[29] X. Jiao, E. E. L. Tanner, S. V. Sokolov, R. G. Palgrave, N. P. Youngc, R. G. Compton, *Phys. Chem. Chem. Phys*., **2017**, *19*, 13547.

[30] T. Matsumoto, W. Nowacki, Zeitschrift für Kristallographie - Crystalline Materials **1966**, *123*, 401-421.


## Author Contributions

**Mahnaz Azimzadeh Sani**: setting up, running and analyzing experiments, particle characterization, drafting manuscript and preparing graphs

**Nicholas G. Pavlopoulos**: Pt nanoparticle synthesis and characterization, contributed to manuscript preparation

**Simone Pezzotti**: interpretation of modelling results, contributed to manuscript preparation and simulation graphics preparation

**Alessandra Serva**: setting up, running and interpretation of modelling results, contributed to manuscript preparation and simulation graphics preparation

**Paolo Cignoni**: Au nanoparticle synthesis and characterization, contributed to manuscript preparation

**Julia Linnemann**: analysis and interpretation of electrochemical results, linking experimental to theory data, contributed to manuscript and graphics preparation

**Mathieu Salanne**: analysis and interpretation of modelling results, contributed to manuscript preparation

**Marie-Pierre Gaigeot**: interpretation of modelling results, contributed to manuscript preparation

**Kristina Tschulik**: development of measurement setup, interpretation of experimental data and link to theory, project planning and management, contributed to manuscript
